# Intersexual and Intrasexual Differences in Mate Selection Preferences Among Lesbian Women, Gay Men, and Bisexual Women and Men

**DOI:** 10.1007/s10508-023-02665-9

**Published:** 2023-08-09

**Authors:** Lisa Klümper, Manfred Hassebrauck, Sascha Schwarz

**Affiliations:** Social Psychology and Personality Psychology, School for Human and Social Sciences, University of Wuppertal, Gaußstraße 20, 42119 Wuppertal, Germany

**Keywords:** Partner preferences, Sexual orientation, Lesbian women, Gay men, Bisexual, Heterosexual

## Abstract

Sex differences in mating strategies and partner preferences are well established. However, most research solely focused on heterosexual women and men. We examined the mate selection, marriage, and age preferences of a sample of lesbian women, gay men, and bisexual women and men (LGB) who took part in an online dating survey. Additionally, we analyzed inter- and intrasexual differences in these preferences. A total of 710 participants rated the importance of 82 mate selection criteria and 10 marriage criteria, and they also indicated their age preferences and short-term and long-term relationship orientation. An exploratory factor analysis suggested 11 relevant domains of mate selection in the LGB sample, with sex, age, and long-term relationship orientation being relevant predictors of differences in these domains. We compared the LGB data with data collected from 21,245 heterosexual women and men on the same mate selection criteria. Results showed that the participants’ sex was the most important predictor of differences in mate selection and marriage preferences, while intrasexual variables (sexual orientation and relationship orientation) explained only a small part of the variance. We incorporated the results into the current discussion about partner preferences and sexual orientation.

## Introduction

For more than 100 years, human mate selection has been intensively studied across the globe (for a historical overview cf. Schwarz & Hassebrauck, 2012). However, a closer look reveals a major gap in the literature, as almost all studies are restricted to heterosexual/straight individuals. Comparatively, only a few studies with limited sample sizes or a limited set of questions addressed a fuller range of diversity in sexual orientation or compared mate choice preferences with the same set of items with the well-known sample of heterosexual/straight individuals. The aim of this current study is to close this major gap in the literature, covering a broader understanding of the potential effects of sexual orientation on mate preferences.

According to a definition from the American Psychological Association (2008), “Sexual orientation refers to an enduring pattern of emotional, romantic and/or sexual attractions to men, women or both sexes. Sexual orientation also refers to a person's sense of identity based on those attractions, related behaviors, and membership in a community of others who share those attractions …” Many studies, especially in mate choice research, rely on individuals self-identifying as “heterosexual/straight” (attracted only to people of the opposite sex), “homosexual/gay/lesbian” (attracted only to people of the same sex), or “bisexual” (attracted to both the same and opposite sex; Frederick et al., 2023). These labels are broad and may not accurately reflect the full range of an individual’s attractions and behaviors and their potential fluctuation across situations and time (Frederick et al., 2023). However, in this paper, we used the self-reported category-based identities to focus on the preferences of lesbian women, gay men, bisexual women and men (LGB) compared to heterosexual/straight men and women in the endeavor to choose a potential partner.

First, we review evidence for sex differences and similarities in human mate choice, starting with a brief overview of mate choice preferences from heterosexual individuals (for a recent review, cf. Buss & Schmitt, 2019; for a historical review, cf. Schwarz & Hassebrauck, 2012), and then focusing on the mate preferences of LGB people. Then, we briefly review interindividual differences in mating strategies beyond sex in heterosexuals (for a recent review cf. Schwarz et al., 2020) and summarize the (sparse) literature on interindividual differences in mating strategies in LGB people.

### Sex Differences and Similarities in Human Mate Choice

A considerable body of literature on heterosexual mate choice dealt with the partner preferences of men and women and has revealed some clear intersexual (between-sex) differences in human mating (for a historical review, cf. Schwarz & Hassebrauck, 2012). One of the first studies on human partner preferences found that women preferred ambition, industriousness, education, general intelligence, and good financial prospects more than men. In contrast, for men, good looks, a desire for home life and children, and being a good cook and housekeeper were more important mate choice characteristics (Hill, 1945). A more recently online dating study with 21,245 single participants replicated this pattern. Women preferred characteristics of a potential male partner which signal that the partner is a good provider (e.g., wealthy and generous, intellectual, sociable, reliable, similar, interpersonal warm) more than men prefer these characteristics in a female partner (Cohen's *d* ranging from 0.24 to 0.79). In contrast, men valued the physical attractiveness of a potential partner as more important than women (*d* = − 0.34; Schwarz & Hassebrauck, 2012). Sex differences in mate preferences seem to be replicable despite some cultural variations in effect sizes (Conroy-Beam et al., 2015; Walter et al., 2020), and, for example, on the relative decrease of importance of some mate choice criteria like chastity through the decades (Buss et al., 2001).

Following an evolutionary perspective, the sex differences in mate preferences result from the constraints of reproductive success (Trivers, 1972), which leads to an asymmetrical minimum parental investment. As men and women have to deal with different reproductive risks, this results in a set of sex-specific mating strategies (for a social role perspective, cf. Wood & Eagly, 2012). For men, finding a fertile mate and getting sexual access is a critical adaptive problem for their reproductive success. As such, on average, evolution should have favored males' short-term mating strategies. However, for women, reproductive success was facilitated by finding reliable mates with resources and a willingness to invest these resources, and therefore, women's long-term mating strategies, on average, were favored (for an overview, cf. Buss & Schmitt, 1993, 2019). These strategies are not fixed or universal, and individuals may use different combinations of short-term and long-term strategies at different points in their lives, and the specific strategies may vary depending on a variety of factors, like mate value, social context, or variations in the environment (Batres, 2020). For example, in cultural contexts where food scarcity was common (e.g., Kenya, Uganda or parts of Ecuador), men preferred relatively heavier women with more body fat than WEIRD countries, such as the US (Sugiyama, 2005). Moreover, men preferred heavier women under harsh economic circumstances (Pettijohn & Jungeberg, 2004), when they felt poor (Nelson & Morrison, 2005), and even when they were hungry (Pettijohn et al., 2009; Swami & Tovée, 2006). These relationships were also observed within the same country in different regions: in safe, economically advantaged areas, men were more likely to prefer slimmer women than in economically less developed regions (Sobal & Strunkard, 1989). One explanation, based on life history theory (Crawford & Anderson, 1989; Kaplan & Gangestad, 2005), proposes that women might actively slow down their reproductive strategy under ecologically safe conditions, for example, by reducing their energy balance through dieting. Under economically insecure conditions, however, women tend to increase their energy balance by calorie intake to accelerate reproduction (Hill et al., 2013, 2014; Salmon et al., 2008). Thus, in humans, as well as in any other animals, life history and ecological factors also shape mate preferences and behaviors.

This research on intersexual differences in mating strategies and partner preferences only looked at heterosexual samples and female-male mating contexts. However, the number of people that are not exclusively heterosexual is not negligible. For example, the percentage of people that identify as lesbian women, gay men, bisexual women and men or transgender (LGBT) is around five to seven percent in different samples (4.5% in the USA in 2017, Newport, 2018, and 5.9% in the EU and 7.4% in Germany in 2016, Deveaux, 2020) and the number of male-male or female-female relationships and marriages have steadily increased recently (e.g., 142,000 same-sex couples were reported in Germany in 2019, Statistisches Bundesamt, 2020).

One might assume that regarding partner preferences, as the evolutionary theorizing for mate selection and mating strategies focuses on reproductive success as the main reason for sex differences, it is difficult to transfer these explanations and predictions to LGB mating strategies, as the sexual recreation and reproductive success may not be as pronounced in comparison to heterosexual couples (Savolainen & Lehmann, 2007; Schwartz et al., 2010). However, recent research suggested that the same evolved mechanisms shaping heterosexual mate preferences might also explain sex differences in mate preferences independent of sexual orientation.

### LGB and Heterosexual Partner Preferences: Sex Differences Independent from Sexual Orientation

Empirically, only a few studies examined partner preferences and mating strategies in not exclusively heterosexual samples (for an overview, see Frederick et al., 2023). Some of these studies found similar mate preferences among heterosexual, gay, and bisexual men, and also when comparing heterosexual women to lesbian and bisexual women (Howard & Perilloux, 2017; Lippa, 2007). It was argued that this pattern suggests that some cognitive mechanisms, evolved through human evolution, are closely tied to biological sex. Therefore, the mate choice criteria are universal across sexual orientations and the only variation is in the desired sex (Howard & Perilloux, 2017).

For example, gay and heterosexual men preferred younger partners and valued their physical attractiveness (Bailey et al., 1994; Conway et al., 2015; Silverthorne & Quinsey, 2000). Both groups showed a similar pattern in rating the attractiveness of highly fertile and lowly fertile women indicating that fertility might be an important cue for perceiving female attractiveness for both gay and heterosexual men (Rinn et al., 2020). As fertility is seen as a cue of reproductive value, it might be theoretically important only to opposite-sex mating contexts. Both groups of men show a similar pattern, which supports the idea that mating is closely tied to biological sex (Howard & Perillux, 2017).

With regards to mate preferences, lesbian and heterosexual women preferred a partner with high status (Ha et al., 2012), pleasant personality (Peplau, 2001), and reliability (Lippa, 2007), and they reported the same level of emotional attachment to a casual sex partner (Howards & Perilloux, 2017). Lesbian women also reported the same interest in uncommitted sex, sexual and emotional jealousy, the importance of a partner's physical attractiveness, sociosexuality, and preference for a younger partner than heterosexual women. Lesbian women showed a larger interest in visual sexual stimuli for lesbian women compared to heterosexual women, and heterosexual women were more concerned with partner status than lesbian women (Bailey et al., 1994). The mate preferences of both lesbian and heterosexual women are context-dependent. For example, lesbian and heterosexual women both value physical attractiveness more for a potential partner in short sexual interactions (Lucas et al., 2011). While for lesbian and heterosexual women, a potential partner's physical attractiveness was not unimportant (Bailey et al., 1994), physical appearance was more important for gay and heterosexual men (Ha et al., 2012).

### LGB and Heterosexual Partner Preferences: Effects of Sexual Orientation Independent from Sex

However, studies also found effects of sexual orientation independent of sex. Gay men and lesbian women valued the sincerity of a potential partner more than heterosexuals (Smith et al., 2011) and displayed this more in personal advertisements (Gonzales & Meyers, 1993; Groom & Pennebaker, 2005). Regarding age preferences, while gay men and lesbian women showed similar patterns to heterosexual men and women, they seemed even less selective than heterosexuals, as they accepted a wider age range when selecting potential partners (Conway et al., 2015; Kenrick et al., 1995). Considering sex typicality, gay men and lesbian women preferred more sex-typical partners than heterosexual men and women (Bailey et al., 1997).

### LGB and Heterosexual Partner Preferences: Sex × Sexual Orientation Interactions

There are also some interactions of sex and sexual orientation reported in the literature. Gay men preferred potential partners that were very masculine in appearance and how they act, whereas lesbian women preferred their partners to have a feminine appearance but not a feminine way of acting (Bailey et al., 1997). Gay men also reported more actual sexual encounters than heterosexual men (Bailey et al., 1994; Gobrogge et al., 2007), and they showed a greater preference for a wealthy and honest partner than heterosexual men (Lippa, 2007). Only a few differences between lesbian and heterosexual women were identified in the literature. For example, lesbian women found financial resources less important than heterosexual women (Smith et al., 2011), and they found both the attractiveness of a partner and their own physical appearance less important than heterosexual women or men (Bailey et al., 1994; Gonzales & Meyers, 1993; Smith & Stillman, 2002).

### LGB and Heterosexual Partner Preferences: Bisexuality and Partner Preferences

Regarding bisexual partner preferences, even fewer data are available. Results from a recent paper combining eight studies on bisexual arousal patterns from US, UK, and Canada suggest that the arousal patterns of bisexual, gay men, and heterosexual men are different from each other. Bisexual men showed stronger genital and subjective arousal than the other two groups of men (Jabbour et al., 2020). More bisexual women than lesbian or heterosexual women offered and requested physical attributes (Smith & Stillmann, 2002). Some results suggest that bisexuals (as opposed to lesbian women, gay men, or heterosexual women or men) reported a higher sex drive and soughed greater sexual excitement. Bisexual women, in particular, reported a higher sex drive and more sexual encounters (Lippa, 2007; Schmitt, 2005; Stief et al., 2014). Safron et al. (2018) compared neural correlates of responses to erotic pictures and videos and found that lesbian women's subjective and neural responses showed greater bias toward females than heterosexual and bisexual women.

To summarize, there is clear evidence for sex differences in mate preferences. However, differences between sexual orientations and interactions between sex and sexual orientations are rather small in effect sizes. There is tremendous variability within each sexual orientation group but less between these groups. Therefore, other variables, like interindividual differences in mating motivation, could shed more light on within-group differences regarding mate preferences.

### Beyond Sex Differences: Intrasexual Mating Strategies

Recent research on mating strategies demonstrated important intrasexual (within-sex) variations in mating strategies and interindividual differences beyond sex (Hallam et al., 2018; Schwarz et al., 2020). Sexual strategies theory and the strategic pluralism model (Gangestad & Simpson, 2000) predict that mating strategies are a flexible set of both long-term and short-term strategies and that especially women (on average) are more open to long-term strategies than men to facilitate their reproductive success, whereas men (on average) are more open to short-term strategies than women. These mating strategies are highly context-dependent, and variation may depend on, for example, the sex ratio in the relevant mating pool, the strategies of rivals in the mating pool, and the costs, which may depend on pursuing one strategy. As such, under some circumstances, men pursue long-term strategies over short-term strategies, increasing the chance of fatherhood and the child's survival (Buss & Schmitt, 2019). Additionally, short-term mating strategies serve some adaptive advantages for women. For example, they can test a partner for a long-term relationship or benefit immediately from a short-term mate's physical strength, genetic quality or economic resources (Buss & Schmitt, 2019; Buss et al., 2017; Greiling & Buss, 2000).

Research indicates interindividual differences in the tendency to pursue short-term or long-term mating strategies. The (one-dimensional) sociosexual orientation*,* as the willingness to engage in uncommitted sexual encounters (Penke & Asendorpf, 2008; Simpson & Gangestad, 1991) and relationship orientation, as the preference for either a short-term or long-term relationship, describe these tendencies and acknowledge that an individual can show preferences for both short-term and long-term relationships, suggesting a two-dimensional model of mating strategies (Herzberg et al., 2022; Schwarz et al., 2008).

Both the sociosexual orientation and relationship orientation can explain additional variations in mate selection preferences and behavior, at least for heterosexual men and women. For example, a long-term relationship orientation strongly predicted the importance of a kind and family-orientated partner. On the other hand, a short-term relationship orientation strongly predicted the importance of a partner's physical attractiveness, independent of sex in a heterosexual sample (Schwarz et al., 2020).

Interestingly, very few studies looked at intrasexual mating strategies in LGB samples. In one study, gay men reported a higher sociosexual orientation than heterosexual men on the behavioral subscale of the SOI, but gay men and heterosexual men did not differ regarding the psychological subscale of the SOI (Bailey et al., 1994). Howard and Perilloux (2017) found that gay men scored higher than heterosexual men on the SOI-R, and this effect was primarily driven by the subscale sociosexual desire (Howard & Perilloux, 2017). Bisexuals scored higher on the SOI-R scales than heterosexuals or gay males or lesbian women, and this effect of sexual orientation was most pronounced in bisexual women when compared to heterosexual and lesbian women (Schmitt, 2005).

However, if and how these interindividual differences in mating strategies relate to mate preferences, and particularly if these intrasexual differences are more (or less) important than biological sex, has not yet been studied in LGB samples.

### The Present Research: Dimensions of Mate Preferences

Differences in mating strategies and partner preferences are well studied, can be replicated, and are robust across the last decades and around the globe. However, empirical research mainly referred to heterosexual participants.

Partner preferences of people with other sexual orientations were often examined by analyzing personal advertisements (Bailey et al., 1997; Conway et al., 2015) or presenting descriptions of potential partners that vary in some characteristics (Bailey et al., 1997; Ha et al., 2012). A different approach is presenting a set of partner characteristics to participants and analyzing the differences in the importance ratings in several areas (Lippa, 2007; Regan et al., 2001).

In one of the first studies that focused exclusively on lesbian women and gay men, Regan and colleagues (2001) studied the preferred partner characteristics for a short- and a long-term relationship of a small sample (*n* = 80) by presenting them with 25 characteristics which had been used in previous (heterosexual) partner preference research. Half of the sample rated the characteristics for a short-term partner and the other half for a long-term partner, splitting the relatively low sample size even more. From a principal component analysis (PCA), they extracted six important domains of mate preference (social status, physical appeal, family orientation, intellectuality, interpersonal sensitivity, expressiveness/responsiveness) and discovered sex differences and differences between short- and long-term relationships. Regan et al. (2001) found sex differences between lesbian women and gay men regarding partner preferences. For example, women showed a greater preference for family orientation in a partner, which was more important for women in a long-term partner than for men. This study revealed the first insights into the partner preference of individuals beyond well-studied heterosexual individuals. However, the robustness and reliability of the results must be replicated as the number of mate choice criteria was restricted (*n* = 25), and there was a small sample size (20 per sexual orientation), and the stability of results in a PCA increases with the higher number of participants.

On the one hand, these results support the notion that men and women across different sexual orientations show similar patterns. However, there is also evidence for the effects of sexual orientation (independent of sex) and meaningful sex × sexual orientation interactions. It is unclear which variable is more important, especially regarding partner preferences. Furthermore, other critical intrasexual differences beyond sex should also be considered when predicting mate preferences. Recent research indicated that some partner preferences, at least in heterosexual mate choice, were better predicted by individual differences in relationship orientation than by sex (Schwarz et al., 2020).

### The Present Research: Goals and Hypotheses

We aimed to gain a holistic picture of the mate preferences of lesbian women, gay men, and bisexual women and men, taking intersexual (sex of participants) as well as intrasexual differences (short-term and long-term relationship orientation) into account. Therefore, we examined the evaluation of 82 mate selection criteria, age preferences, and ten marriage criteria in a big sample of single LGB participants.

First, we focused on relevant domains of mate selection preferences by examining the data from single LGB women and men. As prior research indicated that some of the partner preferences (e.g., for a physically attractive partner or high-status partner) are comparable across participants with different sexual orientations (Ha et al., 2012; Lippa, 2007), we hypothesized (H1) that the structure across the 82 mate selection criteria in the LGB sample is comparable to the preferences of heterosexual people.

Second, we analyzed intersexual and intrasexual differences in relevant domains of mate selection criteria, age preferences, and marriage criteria (Schwarz & Hassebrauck, 2007; Schwarz et al., 2020). Based on prior literature, we expected (H2) sex differences regarding the domains of mate selection criteria. Women, more than men, should prefer characteristics of a potential partner signaling the partner to be a good provider, resulting in higher preferences for signs of wealth, status, and a caring personality (e.g., reliability, trustworthiness, intelligence; H2.1). Men, more than women, should prefer characteristics associated with the physical attributes of a potential partner (e.g., physical attractiveness, youth/younger age; H2.2). For the age preferences (H3), we expected women to accept a higher age for a potential partner than men (H3.1), whereas men should accept a younger age for a potential partner (H3.2).

For the marriage criteria (H4), we expected a similar pattern of preferences for women and men, so women should imagine marrying a partner more likely when showing signs of good wealth, status, and reliability (H4.1). In contrast, men should imagine marrying a partner more when there are signs of youth and attractiveness (H4.2).

Also, as no paper had considered intrasexual differences in mating motivation to qualify differences depending on sexual orientation, we explored potential differences. We assumed that comparable to the results based on heterosexual samples only (e.g., Schwarz et al., 2020), long-term and short-term relationship orientation may be a better predictor for some domains than sex and sexual orientation. Long-term relationship orientation should be a strong predictor for criteria signaling a reliable and trustworthy partner which good providing qualities (E1). In contrast, short-term relationship orientation should be a better predictor for criteria signaling a potential partner's physical aspects (e.g., physical attractiveness; E2).

Finally, we compared the mate selection preferences of lesbian women, gay men, bisexual, and heterosexual women and men to draw some conclusions about the patterns of preferences across sexual orientations.

Besides these assumed sex differences mentioned above, we expected a difference in age preferences depending on sexual orientation. For gay men and lesbian women, we predicted that they accept a wider age range for a potential partner than heterosexual men and women (H5).

Considering potential sex × sexual orientation interactions (H6), we predicted that gay men should prefer a wealthier partner than heterosexual men (H6.1). For women, heterosexual women should prefer wealth and physical attractiveness more than lesbian women (H6.2).

Evidence for the preferences of bisexual persons was quite rare, and there was a systematic lack of evidence. Therefore, we explored potential differences compared to heterosexual, gay men, and lesbian women.

## Method

### Participants

A total of 23,935 participants took part in an online survey which was conducted to examine human mate choice and hosted and advertised by a large German online dating service. For our first and second aims of the study, we took a closer look at participants who self-identified as lesbian women, gay man, or bisexual women or men (*n* = 887). A total of 847 participants completed the whole questionnaire. In addition, we excluded six participants with non-valid responses (e.g., age 200 years) and one that was under the age of 18. As relatively few adults over 65 years participated, these participants were also excluded (*n* = 15) to allow direct comparison with the age range of the heterosexual subsample (see below). Finally, 115 participants indicated that they were in a relationship or married. To avoid possible confounds with relationship status, we excluded these participants too. The final sample for our first analysis (LGB sample) consisted of 710 participants (458 women: 328 bisexuals, 130 lesbian women; 252 men: 157 bisexuals, 95 gay men) aged between 18 and 65 (*M* = 39.19, *SD* = 11.47) and who stated that they were not in a relationship. Participants were well-educated as only 10 participants reported no formal education, 157 reported having their lower secondary school leaving certificate (“Hauptschulabschluss”), 268 had their intermediate secondary school leaving certificate (“Mittlere Reife/Fachhochschulreife”), 111 had their higher secondary school leaving certificate (“Allgemeine Hochschulreife/Abitur”), and 164 had a university degree.

Using G*Power 3.1 (Faul et al., 2009), we conducted a statistical sensitivity analysis (alpha = 0.05, power = 0.80, two-tailed) and compared our results with data from Regan et al. (2001). These analyses showed that Regan et al. (2001) could only detect effect sizes with *d* > 0.63.

Our sample size (95 gay men and 130 lesbian women) allowed us to detect significant effects with a smaller effect size (*d* > 0.38). Finally, our large sample size of 485 bisexual individuals allowed us more profound insights into potential differences between gay men, lesbian women, male and female bisexuals.

Finally, we used data from Schwarz and Hassebrauck (2012) to compare the effects of sex differences in partner preferences in the LGB and heterosexual samples. The sample consisted of 21,245 heterosexual participants (*M* = 41.16, *SD* = 10.54) aged between 18 and 65 (see the method section of Schwarz & Hassebrauck, 2012 for more details). The prior paper already analyzed the mate choice preferences of the heterosexual sample but did not include the data of the LGB participants. To compare the mate choice preferences of LGB and heterosexual participants, we reanalyzed the Schwarz and Hassebrauck’s data (2012) and calculated new scales of common important preferences to directly compare the differences between LGB and heterosexual participants in common mate choice domains.

### Measures

#### Mate Selection Criteria

Participants rated the importance of 82 mate selection criteria for a long-term partner on a five-point scale ranging from 1 = *unimportant* to 5 = *very important*. Examples of the characteristics are seen in Table 1. These criteria were based on a previous prototype analysis of partner preferences (Storz, 2001) and were the same as used in the heterosexual sample reported by Schwarz and Hassebrauck (2012).

**Table 1:** Results of the exploratory factor analysis (EFA) for the domains of mate selection preferences of lesbian women, gay men, and bisexual women and men

	I. Caring	II. Adventurous	III. Enlightened	IV. Cultivated	V. Physically attractive	VI. Wealthy and generous	VII. Approachable	VIII. Comedic	IX. Domestic	X. Like-minded	XI. Child-friendly
Affectionate	**.92**										
Lovingly	**.90**										
Warmhearted	**.73**										
Emotional	**.72**										
Empathic	**.68**										
Considerate	**.58**										
Understanding	**.58**										
Honestly	**.58**										
Faithful	**.49**										
Attentive to my needs	.47										
Responsible	**.46**										
Gives me security	.43										
Well-balanced	.41										
Spontaneous		**.78**									
Flexible		**.70**									
Venturesome		**.66**									
Ambitious		.63									
Outgoing		**.62**									
Self confident		**.60**									
Assertive		**.60**									
Goal orientated		**.57**									
Willing to take risks		**.56**									
Has a mind of his/her own		**.52**									
Individual		**.44**									
Autonomous		**.42**									
High level of education			**.81**								
Educated			**.76**								
Literate			**.68**								
Intelligent			**.67**								
Ingenious			**.66**								
Tolerant			.46								
Critical			**.42**								
Neat				**.71**							
Polite				**.64**							
Industrious				**.59**							
Has good manners				**.59**							
Well-dressed				.48							
Well-tended				.45							
Reliable				.45							
Sexy looks					**.86**						
Attractive					**.78**						
Good looks					**.71**						
Erotic					**.61**						
Exciting					.41						
Rich						**.82**					
Wealthy						**.82**					
Generous						**.49**					
Successful in his/her career						**.46**					
Has a high status						**.45**					
Straightforward							**.56**				
Friendly							**.53**				
Kind							.49				
Pleasant							**.41**				
Funny								**.74**			
Witty								**.68**			
Humorous								**.44**			
Musical									**.49**		
Homebody									**.46**		
Good cook									**.46**		
Similar in interests										**.66**	
Similar in opinions										**.59**	
Similar ideas of a relationship										**.51**	
Wants children											**.72**
Fond of children											**.63**

Items with loadings higher than .40 are included. Bold items are used for the comparison of the LGB and heterosexual sample

#### Age Preferences

The measurement of age preferences was adopted from Hill (1945). Participants indicated in an open text field the lowest (“How many years younger than you should a partner be at most?”) and highest (“How many years older than you should a partner be at most?”) age in years that they would accept for a potential long-term partner.

#### Marriage Criteria

The willingness to marry a potential long-term partner when presenting specific criteria was assessed using 10 marriage criteria adopted from Sprecher et al. (1994). On a dichotomous scale (yes/no), participants indicated if they could imagine marrying someone who, for example, earns more than they do (see Table 5 for all 10 criteria).

#### Relationship Orientation

Individual differences in the participants' long-term and short-term relationship preferences were assessed with the relationship orientation questionnaire (Schwarz & Hassebrauck, 2007; Schwarz et al., 2008; Schwarz et al., 2011). Two subscales demonstrate the preferences for a long-term relationship (α = 0.79; sample item: “Warmth and security are essential parts of a relationship”) and for a short-term relationship (α = 0.87; sample item: “When there is the opportunity, I would like to have sex with as many people as I can”). Participants rated their agreement to the statements on a five-point scale ranging from 1 = *do not agree at all* to 5 = *fully agree*.

#### Personal Information

Participant's age in years (“Please indicate your age here”), sex (male, female), relationship status (single, in a relationship, married, divorced, widowed), and their highest education (without a degree, secondary school diploma, middle school maturity, high school graduation, study degree) were assessed. The participant's sexual orientation was assessed with a self-identified categorical question “Please indicate your sexual orientation” (heterosexual, gay/lesbian, bisexual).

### Procedure

The users of an online dating portal received an email from the dating website advertised as a partner preference study. The participants received the information that the survey would be used to adjust the website optimally to the requirements, and that the survey is anonymous, that there is no comparison with any other personal information on the website, and that their participation is free and not mandatory. After a short introduction on the aims of the study, participants had to rate the mate selection criteria, followed by the age preferences and the marriage characteristics. Participants then completed the relationship orientation questionnaire and indicated their personal information.

## Results

First, we only analyzed responses from the participants who identified as lesbian women, gay men, or bisexual women or men when analyzing inter- and intrasexual differences in LGB people. Therefore, the first analyses were conducted with the LGB sample (*n* = 710) and done with IBM SPSS Statistics 29.

In the second part of the analysis, we compared the mate selection preferences of the LGB sample with the heterosexual sample (i.e., self-identified heterosexual individuals reported in Schwarz & Hassebrauck, 2012). R (Version 4.2.3) was used to compare LGB people in our sample with heterosexual men and women. As the sample sizes of the heterosexual (*n* = 21,245) and the LGB sample (*n* = 710) differed tremendously, the “robust” package (Wang et al., 2020) and “robustbase” package (Maechler et al., 2021; Todorov & Filzmoser, 2009) were used to conduct robust regression analyses for this comparison. Due to the participants' broad age range, age served as a covariate in all analyses.

### Domains of LGB Mate Selection Preferences

We hypothesized that the structure across the 82 mate selection criteria in the LGB sample is comparable to the preferences of heterosexual people (H1). In this section, we first focus on the LGB sample solely. We refer to the direct comparison between the LGB and the heterosexual data later in the results section.

First, we examined the domains of mate selection preferences in the LGB sample (*n* = 710) using an Exploratory Factor Analysis (EFA) with Promax rotation. The Kaiser–Meyer–Olkin and the Bartlett test showed an adequate fit for an EFA (KMO = 0.949, χ^2^_3321_ = 29,385.03, *p* < .001). The parallel analysis suggests the extraction of eleven factors (for the 12th factor: Eigenvalue = 0.40, random Eigenvalue = 0.43).

As can be seen in Table 1, 13 mate selection preferences loaded high onto Factor I. and signaled a preference for a *caring* partner (affectionate, lovingly, warmhearted, emotional, empathic, considerate, understanding, honestly, faithful, attentive to my needs, responsible, gives me security, well-balanced; α = 0.89). Factor II., *adventurous* partner, included 12 items (spontaneous, flexible, venturesome, ambitious, outgoing, self-confident, assertive, goal orientated, willing to take risks, has a mind of his/her own, individual, autonomous; α = 0.88). Factor III. represented a preference for an *enlightened* partner and included seven items (high level of education, educated, literate, intelligent, ingenious, tolerant, critical; α = 0.85). Factor IV., a *cultivated* partner, included seven items (neat, polite, industrious, has good manners, well-dressed, well-tended, reliable; α = 0.83). Factor V., a *physically attractive* partner, included five items (sexy looks, attractive, good looks, erotic, exciting; α = 0.81). Factor VI., a *wealthy and generous* partner, included five items (rich, wealthy, generous, successful in their career, has a high status; α = 0.82). Factor VII., an *approachable* partner, included four items (straightforward, friendly, kind, pleasant; α = 0.76). Factor VIII., preferences for a *comedic* partner consisted of three items (funny, witty, humorous; α = 0.79). The ninth Factor IX. included three items representing a preference for a *domestic* partner (musical, homebody, good cook; α = 0.62). The last two factors, Factor X., representing the preference for a *like-minded partner* with three items (similar in interests, similar in opinions, similar ideas of a relationship; α = 0.74) and Factor XI., preference for a *child-friendly* partner, with two items (wants children, fond of children; α = 0.67; for an overview of descriptive statistics and intercorrelations between the scales, see Table 2 and 3).

**Table 2:** Intercorrelations between the domains of mate selection preferences of lesbian women, gay men, and bisexual women and men

Domains of mate selection preference	I.	II.	III.	IV.	V.	VI.	VII.	VIII.	IX.	X.	XI.
I. Caring	(.89)										
II. Adventurous	.54	(.88)									
III. Enlightened	.43	.58	(.85)								
IV. Cultivated	.63	.54	.42	(.83)							
V. Physically attractive	.28	.44	.29	.31	(.81)						
VI. Wealthy and generous	.26	.45	.46	.40	.31	(.82)					
VII. Approachable	.66	.54	.34	.58	.37	.29	(.76)				
VIII. Comedic	.47	.53	.33	.44	.35	.17	.45	(.79)			
IX. Domestic	.36	.35	31	.36	.27	.44	.37	.17	(.62)		
X. Like-minded	.51	.42	.29	.43	.26	.26	.45	.27	.35	(.74)	
XI. Child-friendly	.31	.25	.13	.20	.17	.17	.20	.23	.24	.23	(.67)

Cronbach’s alphas are shown in parentheses in the diagonal. All correlations are significant at *p* < .001

**Table 3:** Descriptive statistics for the eleven domains of mate selection preferences for lesbian women, gay men, and bisexual women and men based on an exploratory factor analysis on the LGB sample (see Table 1, all factor loadings)

Domains of mate selection preference	Gay men		Lesbian women		Bisexual men		Bisexual women	
	*M*	*SD*	*M*	*SD*	*M*	*SD*	*M*	*SD*
I. Caring	4.18	0.61	4.46	0.44	4.16	0.55	4.35	0.51
II. Adventurous	3.68	0.64	3.85	0.53	3.58	0.60	3.79	0.61
III. Enlightened	3.41	0.74	3.70	0.58	3.51	0.71	3.72	0.68
IV. Cultivated	3.91	0.66	4.18	0.52	3.98	0.72	4.06	0.59
V. Physically attractive	3.92	0.73	3.65	0.68	3.94	0.70	3.71	0.76
VI. Wealthy and generous	2.74	0.83	3.05	0.83	2.60	0.83	2.97	0.85
VII. Approachable	4.01	0.72	4.16	0.62	3.95	0.75	4.12	0.75
VIII. Comedic	4.07	0.63	4.13	0.61	4.05	0.63	4.01	0.67
IX. Domestic	3.07	0.88	2.91	0.82	3.04	0.87	2.86	0.86
X. Like-minded	3.73	0.86	4.11	0.61	3.90	0.78	3.91	0.75
XI. Child-friendly	3.12	1.35	3.38	1.17	3.15	1.14	3.21	1.25

### Intersexual Differences in Mate Selection Criteria (H2)

We computed unit-weighted scales based on the LGB mate selection preference structure. At first, we considered sex differences in the domains of mate selection criteria (see Table 3 for the descriptive statistics). We conducted a two-way MANCOVA with the sex of participants (men vs. women), sexual orientation (gay/lesbian vs. bisexual), and participant's age as a covariate in the LGB sample on the eleven mate selection criteria. There were significant multivariate main effects of sex, Pillai's Trace *V* = 0.214, *F*(11, 695) = 17.25, *p* < .001, part. η^2^ = 0.214, and sexual orientation, Pillai's Trace *V* = 0.038, *F*(11, 695) = 2.48, *p* = .005, part. η^2^ = 0.038, and a significant multivariate sex × sexual orientation interaction, Pillai’s Trace *V* = 0.032, *F*(11, 695) = 2.11, *p* = .018, part. η^2^ = 0.010.

Univariate ANCOVAs revealed sex differences in nine domains: in line with H2.1, women (more than men) showed a higher preference for a caring partner, *F*(1, 705) = 31.13, *p* < .001, part. η^2^ = 0.042, for an enlightened partner, *F*(1, 705) = 22.03, *p* < .001, part. η^2^ = 0.030, and for a wealthy and generous partner, *F*(1, 705) = 28.42, *p* < .001, part. η^2^ = 0.039. Moreover, women showed higher preferences for an adventurous partner, *F*(1, 705) = 12.99, *p* < .001, part. η^2^ = 0.018, for a cultivated partner, *F*(1, 705) = 13.47, *p* = .001, part. η^2^ = 0.019, and for a comedic partner, *F*(1, 705) = 4.73, *p* = .030, part. η^2^ = 0.007, and a like-minded partner, *F*(1, 705) = 10.25, *p* = .001, part. η^2^ = 0.014. Also, according to the expectations (H2.2), men (more than women) showed a significantly higher preference for a physically attractive partner, *F*(1, 705) = 20.91, *p* < .001, part. η^2^ = 0.029. Men (more than women) showed a higher preference for a domestic partner, *F*(1, 705) = 4.15, *p* = .042, part. η^2^ = 0.006. There were no differences for an approachable partner, *F*(1, 705) = 0.27, *p* = .603, part. η^2^ < 0.001, and a child-friendly partner, *F*(1, 705) = 0.13, *p* = .719, part. η^2^ < 0.001.

The univariate ANCOVAs revealed a significant effect for the sexual orientation for only one domain: gay/lesbian persons showed higher preferences for a wealthy and generous partner than bisexual individuals, *F*(1, 705) = 4.88, *p* = .028, part. η^2^ = 0.007, and no other significant differences were found, *F*s < 3.05, *p*s > .081, part. η^2^s < 0.005.

Moreover, the analyses revealed two significant univariate sex × sexual orientation interaction effects, for a like-minded partner, *F*(1, 705) = 8.33, *p* = .004, part. η^2^ = 0.011, and a child-friendly partner, *F*(1, 705) = 7.71, *p* = .006, part. η^2^ = 0.011. All other interactions were not significant, *F*s < 3.18, *p*s > .075, part. η^2^s < 0.005. A closer look at the interactions revealed that for a like-minded partner, gay men showed a significantly lower preference than lesbian women, *t* = − 3.78, *p* < .001*, d* = − 0.51*,* and lesbian women showed a significantly higher preference than bisexual women, *t* = 2.67, *p* = .040*, d* = 0.28*.* No other significant differences occurred between the groups. For a child-friendly partner, gay men showed a lower preference than lesbian women, *t* = − 2.66, *p* = .040, *d* = − 0.35*,* and there were no other significant differences between the groups.

### Intersexual Differences in Age Acceptance (H3)

We analyzed the minimum and maximum partner's age accepted by conducting a two-way MANCOVA with the sex of participants (men vs. women), sexual orientation (gay/lesbian vs. bisexual), and participant's age as a covariate. There were three missing responses for the minimum age, so the analysis was conducted with *n* = 707, and as there were two missing responses for the maximum age, the analysis was conducted with *n* = 708. There was a significant multivariate effect of sex, Pillai's Trace *V* = 0.242, *F*(2, 700) = 111.88, *p* < .001, part. η^2^ = 0.242, but not for sexual orientation, Pillai's Trace *V* = 0.005, *F*(2, 700) = 1.87, *p* = .155, part. η^2^ = 0.005, nor sex × sexual orientation interaction, Pillai's Trace *V* = 0.001, *F*(2, 700) = 0.37, *p* = .693, part. η^2^ = 0.001.

In line with the assumptions (H3.1), women accepted a maximum age about ten years older than themselves (*M* = 10.14, *SD* = 9.38), whereas men would accept a maximum age of seven years older (*M* = 7.21, *SD* = 11.92), *F*(1, 701) = 311.51, *p* < .001. On the other hand (H3.2), men indicated that they would accept a partner about eleven years younger (*M* = 11.20, *SD* = 6.68) than themselves, whereas women would accept a minimum age about five years younger (*M* = 4.97, *SD* = 3.73), *F*(1, 701) = 12.18, *p* < .001.

### Intersexual Differences in the Ten Marriage Criteria (H4)

We analyzed the differences between the sexes in the responses to the ten marriage partner characteristics by LGB people (adopted from Sprecher et al., 1994) with several binomial regression analyses with sex (− 1 for men, 1 for women), sexual orientation (− 1 for gay/lesbian, 1 for bisexual) as predictors and age as a covariate. We used odds ratios (OR) for the interpretation of the results. For sex differences, an OR greater than 1 indicates a higher probability that women will respond to the question with “no”, whereas an OR lower than 1 indicates that men are more likely to respond with “no”.

In all analyses, we found no significant effect of sexual orientation or sex × sexual orientation interaction. We found no significant sex differences for six marriage characteristics (having a higher income, having a higher education, the partner's look, having another religion, having been married already, and having children already). We only found significant sex differences for a partner's lower education, lower earning potential, no regular employment, and different skin color. In line with our expectation (H4.1), when the partner earns much less than they do, more women (57%) cannot imagine marrying this person compared to men (15.9%), *OR* = 0.11, 95% CI [0.71, 0.17], Wald *χ*^2^ = 102.76, *p* < .001. More women (66.4%) compared to men (31.0%) could not imagine marrying someone who has no regular employment, *OR* = 0.22, 95% CI [0.15, 0.31], Wald *χ*^2^ = 71.78, *p* < .001. Women (51.5%) compared to men (21.4%) could not imagine marrying someone with a lower level of education than they have, *OR* = 0.22, 95% CI [0.15, 0.33], Wald *χ*^2^ = 56.69, *p* < .001. Not expected, more women (41.3%) than men (29.4%) could not imagine having a partner with a different skin color, *OR* = 0.51, 95% CI [0.36, 0.73], Wald *χ*^2^ = 13.79, *p* < .001. This supported our hypotheses (H4) only partly.

### Inter- and Intrasexual Differences in Mate Selection Domains (E)

No study so far tested for intrasexual differences in mating strategies for mate preferences in an LGB sample. To exploratory (E) examine whether there are other intrasexual differences and whether intersexual or intrasexual differences are more important when predicting mate selection preferences (e.g., Schwarz et al., 2020), we analyzed the data with several multiple regression analyses. Age served as a covariate in all analyses. For each of the eleven mate selection domains as the dependent variable, we included the sex of participants (− 1 for men, 1 for women), sexual orientation (− 1 for gay/lesbian, 1 for bisexual), both relationship orientation scales (mean-centered long-term and short-term relationship orientation), and the two-way and three-way interactions. The results of these analyses can be found in Table 4. Participants' age was a significant covariate across nine analyses. As age was only considered a covariate and was never the single strongest predictor, it will not be discussed further in the results.

**Table 4:** Results of the regression analyses to predict the preferences for the mate selection domains of lesbian women, gay men, and bisexual women and men

	*B* [95% CI]										
	I. Caring	II. Adventurous	III. Enlightened	IV. Cultivated	V. Physically attractive	VI. Wealthy and Generous	VII. Approachable	VIII. Comedic	IX. Domestic	X. Like-minded	XI. Child-friendly
(Constant)	4.28 [4.24, 4.32]	3.74 [3.68, 3.79]	3.61 [3.55, 3.67]	4.05 [4.00, 4.10]	3.81 [3.74, 3.87]	2.84 [2.76, 2.91]	4.06 [4.01, 4.11]	4.08 [4.02, 4.14]	2.99 [2.92, 3.07]	3.91 [3.84, 3.97]	3.24 [3.17, 3.31]
Age	0.01*** [0.01, 0.01]	0.003 [− 0.001, 0.01]	0.01*** [0.01, 0.02]	0.01*** [0.01, 0.02]	− 0.001 [− 0.01, 0,00]	0.02*** [0.01, 0.02]	0.01*** [0.01, 0.02]	− 0.01* [− 0.01, − 0.001]	0.02*** [0.01, 0.02]	0.01*** [0.01, 0.02]	− 0.03*** [− 0.03, − 0.03]
Sex	0.07*** [0.03, 0.11]	0.09** [0.04, 0.14]	0.13*** **[0.07, 0.19]**	0.06* [0.01, 0.12]	− 0.08* [− 0.14, − 0.02]	0.22*** **[0.14, 0.29]**	− 0.004 [− 0.06, 0.05]	0.06 [− 0.004, 0.12]	− 0.09* [− 0.16, − 0.02]	0.06 [− 0.01, 0.12]	− 0.07 [− 0.14, 0.01]
Sexual Orientation (Sexor)	− 0.02 [− 0.06, 0.02]	− 0.05 [− 0.10, 0.01]	0.02 [− 0.04, 0.08]	− 0.02 [− 0.08, 0.03]	0.02 [− 0.06, 0.07]	− 0.10** **[− 0.17, − 0.02]**	− 0.04 [− 0.09, 0.02]	− 0.002 [− 0.07, 0.06]	− 0.05 [− 0.12, 0.03]	− 0.01 [− 0.08, 0.05]	0.04 [− 0.04, 0.11]
Long-term Orientation (LTO)	0.36*** **[0.31, 0.41]**	0.25*** **[0.18, 0.31]**	0.21*** **[0.14, 0.29]**	0.33*** **[0.26, 0.39]**	0.21*** **[0.14, 0.29]**	0.26*** **[0.16, 0.35]**	0.33*** **[0.26, 0.39]**	0.20*** **[0.12, 0.28]**	0.42*** **[0.33, 0.51]**	0.40*** **[0.32, 0.48]**	1.04*** **[0.95, 1.14]**
Short-term Orientation (STO)	− 0.05* [− 0.09, − 0.01]	0.05 [− 0.01, 0.10]	0.04 [− 0.02, 0.11]	− 0.02 [− 0.07, 0.03]	0.22*** **[0.15, 0.28]**	0.14*** **[0.07, 0.22]**	0.03 [− 0.03, 0.08]	0.03 [− 0.04, 0.10]	0.05 [− 0.03, 0.13]	− 0.03 [− 0.09, 0.04]	− 0.01 [− 0.09, 0.07]
Sex × Sexor	0.01 [− 0.04, 0.05]	0.01 [− 0.04, 0.07]	− 0.03 [− 0.09, 0.03]	− 0.02 [− 0.07, 0.04]	0.003 [− 0.06, 0.07]	0.04 [− 0.03, 0.11]	− 0.01 [− 0.06, 0.04]	− 0.02 [− 0.09, 0.04]	0.01 [− 0.06, 0.09]	− 0.04 [− 0.11, 0.02]	− 0.06 [− 0.13, 0.02]
Sex × LTO	− 0.01 [− 0.06, 0.04]	0.03 [− 0.03, 0.09]	− 0.04 [− 0.11, 0.03]	− 0.07* [− 0.13, − 0.01]	− 0.02 [− 0.10, 0.05]	0.02 [− 0.06, 0.11]	0.03 [− 0.04, 0.09]	− 0.02 [− 0.09, 0.05]	− 0.01 [− 0.09, 0.08]	− 0.05 [− 0.13, 0.02]	0.001 [− 0.09, 0.09]
Sex × STO	− 0.03 [− 0.07, 0.01]	0.03 [− 0.02, 0.09]	0.04 [− 0.02, 0.10]	0.004 [− 0.05, 0.06]	0.01 [− 0.05, 0.08]	− 0.002 [− 0.08, 0.07]	− 0.02 [− 0.07, 0.04]	0.03 [− 0.07, 0.03]	0.04 [− 0.03, 0.12]	− 0.03 [− 0.10, 0.03]	0.03 [− 0.04, 0.11]
Sexor × LTO	0.02 [− 0.03, 0.07]	− 0.02 [− 0.08, 0.03]	− 0.01 [− 0.08, 0.06]	0.06* [0.001, 0.12]	− 0.04 [− 0.11, 0.04]	− 0.08 [− 0.17, 0.01]	0.03 [− 0.04, 0.09]	0.04 [− 0.04, 0.11]	− 0.07 [− 0.16, 0.02]	− 0.05 [− 0.12, 0.03]	− 0.10* **[**− **0.19, − 0.01]**
Sexor × STO	0.01 [− 0.04, 0.05]	− 0.01 [− 0.06, 0.05]	− 0.08** **[− 0.15, − 0.02]**	0.02 [− 0.03, 0.07]	0.01 [− 0.06, 0.07]	− 0.04 [− 0.12, 0.03]	− 0.03 [− 0.08, 0.03]	− 0.04 [− 0.11, 0.09]	− 0.06 [− 0.14, 0.02]	0.05 [− 0.01, 0.12]	0.01 [− 0.06, 0.09]
Sex × Sexor × STO	0.01 [− 0.03, 0.06]	− 0.001 [− 0.06, 0.05]	0.07* [0.01, 0.13]	− 0.02 [− 0.07, 0.04]	0.01 [− 0.05, 0.08]	0.04 [− 0.04, 0.11]	0.03 [− 0.03, 0.08]	0.02 [− 0.05, 0.09]	0.01 [− 0.06, 0.09]	0.03 [− 0.04, 0.09]	− 0.02 [− 0.09, 0.06]
Sex × Sexor × LTO	− 0.02 [− 0.07, 0.02]	0.02 [− 0.04, 0.08]	0.10** **[0.03, 0.17]**	− 0.06 [− 0.12, 0.003]	0.01 [− 0.06, 0.09]	0.09* **[0.01, 0.18]**	− 0.04 [− 0.10, 0.03]	− 0.01 [− 0.09, 0.06]	− 0.03 [− 0.12, 0.05]	0.08* **[0.001, 0.15]**	0.10* **[0.01, 0.19]**

Sex (higher value for women) and sexual orientation (higher value for bisexual) are contrast-coded; LTO, STO, and age are mean-centered. Best predictors (largest predictive value and no CI overlap with other predictors) are printed bold

***p < .001, **p < .01, *p < .05

As shown in Table 4, sex (seven domains) and long-term relationship orientation (all eleven domains) were significant predictors for the mate selection domains. Short-term relationship orientation was a significant predictor for three domains, and sexual orientation for one domain. There was one significant sex × long-term orientation interaction, two significant sexual orientation × long-term orientation interactions, and one significant sexual orientation × short-term relationship orientation interaction. Also, five significant three-way interactions were identified.

**Fig. 1:**
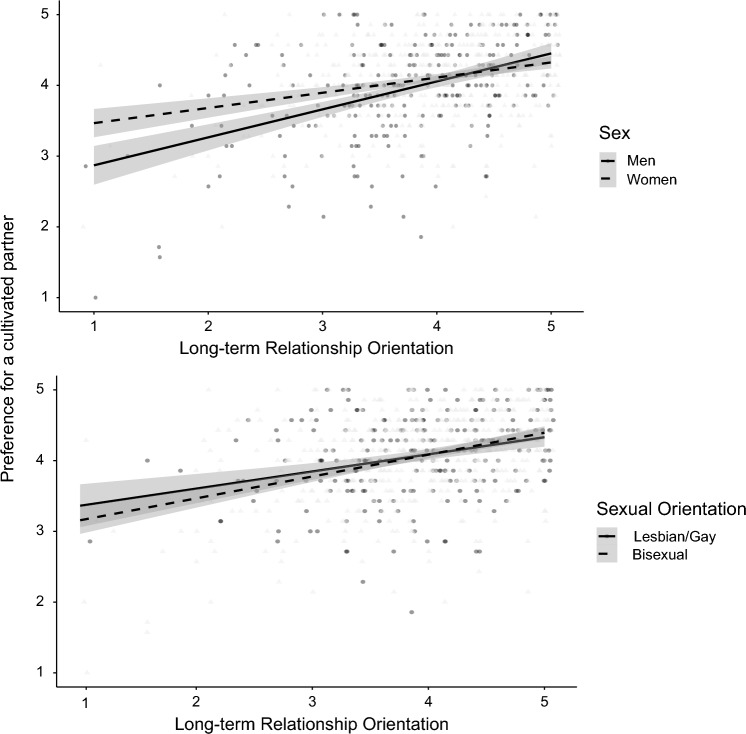
Preference for a cultivated partner as a function of participant's sex and long-term relationship orientation (upper panel), and sexual orientation and long-term relationship orientation (lower panel). LGB sample size *n* = 710. Gray bands represent the 95% CI

For Factor IV., a cultivated partner, there was a significant interaction of sex and long-term relationship orientation (Δ*R*^2^ = .010, *F*(1, 697) = 5.28, *p* = .022), and a significant sexual orientation and long term-relationship orientation interaction (Δ*R*^2^ = .005, *F*(1, 697) = 3.96, *p* = .047). As can be seen in Fig. 1 (upper panel), for both men,* B* = 0.42, *SE* = 0.05, *t* = 8.25, *p* < .001, 95% CI [0.32, 0.52], and women*, B* = 0.29, *SE* = 0.04,* t* = 7.03, *p* < .001, 95% CI [0.21, 0.37], and for lesbian/gay, *B* = 0.24, *SE* = 0.05,* t* = 4.34, *p* < .001, 95% CI [0.13, 0.34], and bisexual people, *B* = 0.36, *SE* = 0.04,* t* = 10.46, *p* < .001, 95% CI [0.29, 0.43], long-term relationship orientation was significantly associated with a preference for a cultivated partner, while the association was steeper for men, and for bisexual people (Fig. 1, lower panel). With higher levels of long-term relationship orientation, the differences between the respective groups vanished.

**Fig. 2:**
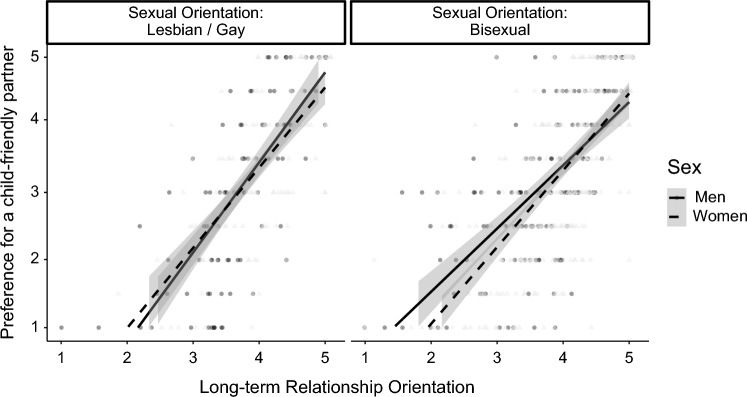
Preference for a child-friendly partner as a function of participant's sex, sexual orientation, and long-term relationship orientation. LGB sample size *n* = 710. Gray bands represent the 95% CI

There was a significant interaction of sexual orientation and long-term relationship orientation for the Factor XI., child-friendly partner, which was qualified by an significant three- way interaction of sex × sexual orientation × long-term relationship orientation (Δ*R*^2^ = .003, *F*(1, 697) = 5.02, *p* = .025; see Fig. 2). Simple slope analyses revealed no significant sex × long-term relationship orientation interaction for gay/lesbian people, *B* = − 0.12, *SE* = 0.07,* t* = − 1.64, *p* = .103, 95% CI [− 0.26, 0.02], but for bisexual people, *B* = 0.10, *SE* = 0.05, *t* = 2.01, *p* = .045, 95% CI [0.002, 0.20]. The relationship for men and long-term relationship orientation, *B* = 0.840, *SE* = 0.08, *t* = 10.42, *p* < .001, 95% CI [0.68, 1.00], and women,* B* = 1.03, *SE* = 0.07, *t* = 15.50, *p* < .001, 95% CI [0.90, 1.17], was significant, and the difference between women and men was not significant with high levels of long-term relationship orientation, *B* = − 0.41, *SE* = 0.06, *t* = − 0.73, *p* = .468, 95% CI [− 0.15, 0.07].

For Factor III., an enlightened partner, there were two significant three-way interactions of sex and sexual orientation with long-term and short-term relationship orientation.

**Fig. 3:**
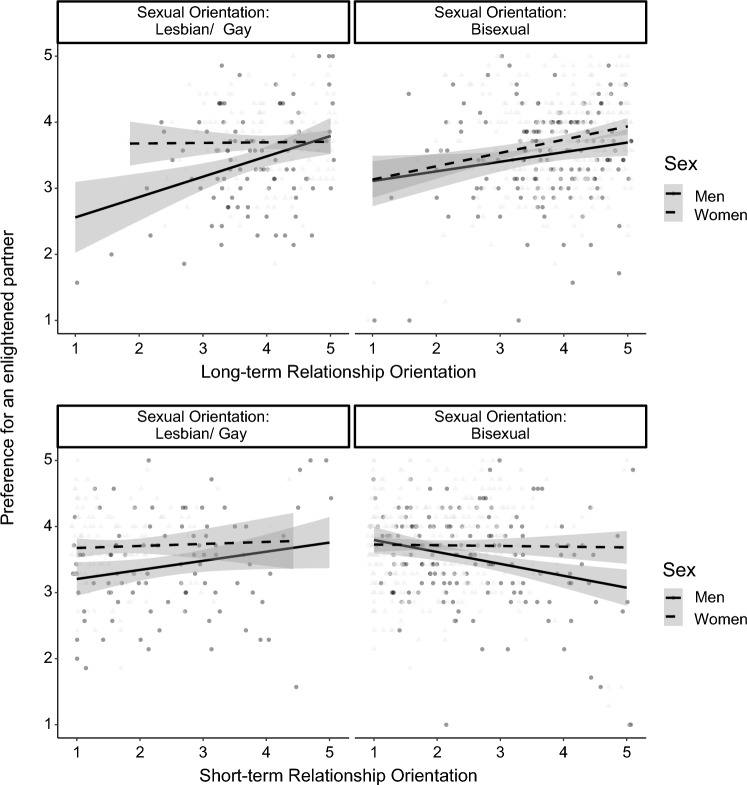
Preference for an enlightened partner as a function of participant's sex, sexual orientation, and long-term relationship orientation. LGB sample size *n* = 710. Gray bands represent the 95% CI

There was no significant sexual orientation × long-term relationship orientation interaction for men, *B* = − 0.11, *SE* = 0.06, *t* = − 1.90, *p* = .059, 95% CI [− 0.22, 0.004], nor for women, *B* = 0.09, *SE* = 0.05, *t* = 1.88, *p* = .060, 95% CI [− 0.004, 0.18]. There was only a significant sex × long-term relationship orientation interaction for gay/lesbian people, *B* =  − 0.14, *SE* = 0.06, *t* = − 2.53, *p* = .012, 95% CI [− 0.25, − 0.02], but not for bisexual people, *B* = 0.06, *SE* = 0.04, *t* = 1.40, *p* = .162, 95% CI [− 0.02, 0.14]. As can be seen in Fig. 3 (upper panel), there was a positive association with long-term relationship orientation for gay men, *B* = 0.36, *SE* = 0.09, *t* = 3.77, *p* < .001, 95% CI [0.17, 0.54], but not lesbian women, *B* = 0.11, *SE* = 0.08, *t* = 1.33, *p* = .187, 95% CI [− 0.05, 0.26].

Regarding the sex × sexual orientation × short-term relationship orientation, there was a significant sex × short-term relationship orientation interaction for bisexual people, *B* = 0.11, *SE* = 0.03, *t* = 3.22, *p* = .001, 95% CI [0.04, 0.17], but not for gay/lesbian people, *B* = − 0.03, *SE* = 0.05, *t* = − 0.54, *p* = .590, 95% CI [− 0.13, 0.07]. Additionally, there was a significant sexual orientation × short-term relationship orientation interaction for men, *B* =  − 0.16, *SE* = 0.04, *t* = − 3.63, *p* < .001, 95% CI [− 0.22, − 0.07], but not for women, *B* =  − 0.02, *SE* = 0.05, *t* = − 0.34, *p* = .733, 95% CI [− 0.10, 0.07]. As can be seen from Fig. 3 (lower panel), for gay men, there was a significant association between short-term orientation and preference, *B* = 0.16, *SE* = 0.07, *t* = 2.37, *p* = .020, 95% CI [0.03, 0.29], and for bisexual men, *B* =  − 0.15, *SE* = 0.05, *t* = − 2.84, *p* = .005, 95% CI [− 0.26, − 0.05].

**Fig. 4:**
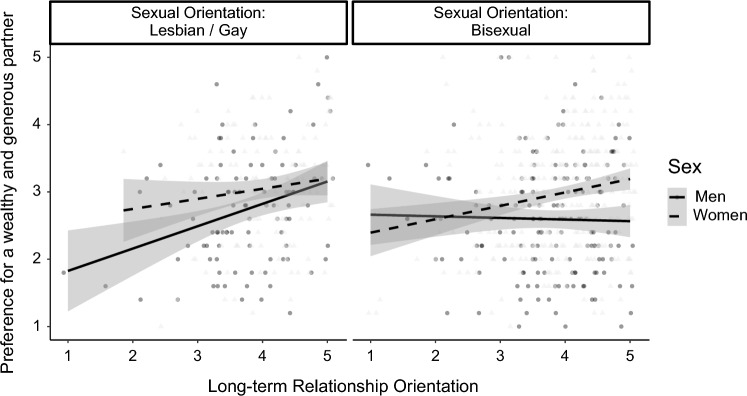
Preference for a wealthy and generous partner as a function of participant's sex, sexual orientation, and long-term relationship orientation. LGB sample size *n* = 710. Gray bands represent the 95% CI

Additional three-way interactions were found for Factor VI., a wealthy and generous partner (Δ*R*^2^ = .009, *F*(1, 697) = 7.02, *p* = .008). As can be seen in Fig. 4, there was no significant sex × long-term relationship orientation interaction for gay/lesbian people, *B* = − 0.09, *SE* = 0.07, *t* = − 1.16, *p* = .248, 95% CI [− 0.23, 0.06], so that the association of long-term relationship orientation was significant for gay men, *B* = 0.36, *SE* = 0.11, *t* = 3.44, *p* < .001, 95% CI [0.15, 0.57], and lesbian women, *B* = 0.23, *SE* = 0.12, *t* = 1.98, *p* = .050, 95% CI [0.001, 0.45]. There was a significant interaction for bisexual people, *B* = 0.12, *SE* = 0.05, *t* = 2.41, *p* = .016, 95% CI [0.02, 0.22]. There was no significant association for bisexual men, *B* = 0.07, *SE* = 0.08, *t* = 0.79, *p* = .431, 95% CI [− 0.10, 0.23], but for bisexual women, *B* = 0.32, *SE* = 0.06, *t* = 5.14, *p* < .001, 95% CI [0.19, 0.44]. The sexual orientation × long-term relationship orientation interaction was only significant for men,* B* = − 0.18, *SE* = 0.07, *t* = − 2.66, *p* = .008, 95% CI [− 0.31, − 0.05], but not for women, *B* = 0.01, *SE* = 0.06, *t* = 0.22, *p* = .829, 95% CI [− 0.10, 0.13].

**Fig. 5:**
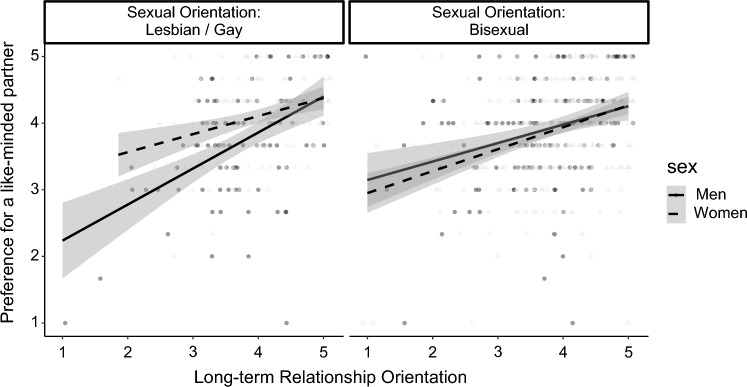
Preference for a like-minded partner as a function of participant's sex, sexual orientation, and long-term relationship orientation. LGB sample size *n* = 710. Gray bands represent the 95% CI

Lastly, a significant sex × sexual orientation × long-term relationship orientation interaction was found for Factor X., a like-minded partner (Δ*R*^2^ = .005, *F*(1, 697) = 3.94, *p* = .048; see Fig. 5). For gay/lesbian people, there was a significant sex × long-term relationship orientation interaction, *B* = − 0.13, *SE* = 0.06, *t* = − 2.23, *p* = .027, 95% CI [− 0.25, − 0.02], but no significant interaction for bisexual people, *B* = 0.03, *SE* = 0.04, *t* = 0.59, *p* = .555, 95% CI [− 0.06, 0.11]. There was a significant sexual orientation × long-term relationship orientation interaction for men, *B* = − 0.12, *SE* = 0.06, *t* = − 2.01, *p* = .045, 95% CI [− 0.25, − 0.003], but not for women, *B* = 0.03, *SE* = 0.05, *t* = 0.57, *p* = .566, 95% CI [− 0.07, 0.12]. Long-term relationship orientation was significantly associated with the preference for a like-minded partner for gay men, *B* = 0.54, *SE* = 0.10, *t* = 5.21, *p* < .001, 95% CI [0.34, 0.75], and bisexual men, *B* = 0.35, *SE* = 0.08, *t* = 4.61, *p* < .001, 95% CI [0.20, 0.50]. As can be seen from Fig. 5, the association between long-term relationship orientation and the preference is steeper for gay men compared to bisexual men.

We used the 95% confidence interval (CI) to identify the strongest predictors in these analyses (printed in bold in Table 4). If two predictors showed an overlap between the CIs, both were viewed as equally strong (printed in bold). Long-term relationship orientation was the single most important predictor in six of the eleven domains. Short-term relationship orientation was important for two domains, but not solely. For the third domain, an enlightened partner, various factors and their interaction were relevant for the prediction. Also, for the fifth domain, a wealthy and generous partner, sex, sexual orientation, and long-term and short-term relationship orientation were seen as equally important predictors.

### Inter- and Intrasexual Differences in Age Acceptance (E)

To examine whether additional intrasexual differences existed and whether intersexual or intrasexual differences were more important when predicting the minimum and maximum age accepted, we conducted two multiple regression analyses—one for the minimum age and one for the maximum age of a potential partner. Again, age served as covariate in all analyses. We included the sex of participants (− 1 for men, 1 for women), sexual orientation (− 1 for gay/lesbian, 1 for bisexual), both relationship orientation scales (mean-centered long-term and short-term relationship orientation), and all two-way and three-way interactions in the models.

For the maximum age, there was again a significant sex difference, *B* = 1.53, *SE* = 0.47, *t* = − 3.25, *p* = .001, 95% CI [0.61, 2.45]. As reported in the previous section, the maximum accepted age for women was higher than for men, as women accept a greater difference to their own age. Moreover, there was a significant effect for sexual orientation, *B* = 0.96, *SE* = 0.48, *t* = 2.01, *p* = .045, 95% CI [0.02, 1.89]. Furthermore, the higher the long-term orientation, the less restrictive the participants was in regard to the maximum age of a partner (i.e., they would accept a wider age range), *B* = − 1.84, *SE* = 0.58, *t* =  − 3.16, *p* = .002, 95% CI [− 2.98, − 0.79]. There was a significant interaction of sex and long-term orientation, *B* = 1.67, *SE* = 0.56, *t* = 3.00, *p* = .003, 95% CI [0.58, 2.77], so for men, long-term relationship orientation was negatively associated with the maximum age accepted, *B* = − 2.60, *SE* = 0.97, *t* = − 2.68, *p* = .008, 95% CI [− 4.51, − 0.69], but not for women, *B* = − 0.18, *SE* = 0.62, *t* = − 0.02, *p* = .769, 95% CI [0.1.39, 1.03]. Also, there was a significant sexual orientation and long-term orientation interaction, *B* = 1.17, *SE* = 0.56, *t* = 2.10, *p* = .037, 95% CI [0.07, 2.26], so for gay /lesbian people, long-term relationship orientation was negatively associated with the maximum age accepted, *B* =  − 3.01, *SE* = 1.02, *t* = − 2.96, *p* = .003, 95% CI [− 5.01, − 1.01], but not for bisexual people, *B* = − 0.38, *SE* = 0.62, *t* =  − 0.62, *p* = .535, 95% CI [− 1.60, 0.83].

There was also a significant sex × sexual orientation × short-term orientation interaction, *B* = 1.54, *SE* = 0.48, *t* = 3.21, *p* = .001, 95% CI [0.60, 2.48]. Post-hoc analyses revealed a significant sex × short-term orientation interaction for gay/lesbian people, *B* = − 2.40, *SE* = 0.83, *t* = − 2.88, *p* = .004, 95% CI [− 4.04, − 0.76], whereas there was no such interaction for bisexual people, *B* = 0.90, *SE* = 0.51, *t* = 1.77, *p* = .077, 95% CI [− 0.10, 1.89]. For both men, *B* = − 1.47, *SE* = 0.72, *t* = − 2.04, *p* = .042, 95% CI [− 2.89, − 0.05], and women, *B* = 1.60, *SE* = 0.66, *t* = 2.44, *p* = .015, 95% CI [0.31, 2.91], there was a significant sexual orientation × short-term orientation interaction. There was a positive association of short-term orientation for gay men, *B* = 3.00, *SE* = 1.23, *t* = 2.45, *p* = .016, 95% CI [0.56, 5.44], and bisexual women, *B* = 1.77, *SE* = 0.63, *t* = 2.81, *p* = .005, 95% CI [0.53, 3.01]. There was no significant association for both bisexual men, *B* = 0.27, *SE* = 0.85, *t* = 0.32, *p* = .749, 95% CI [− 1.40, 1.95], or lesbian women, *B* =  − 1.36, *SE* = 1.07, *t* = − 1.27, *p* = .208, 95% CI [− 3.48, 0.77].

For the minimum accepted age, as reported in the previous section, men would accept a partner significantly younger than themselves more than women would, *B* = − 2.40, *SE* = 0.20, *t* = − 11.89, *p* = .001, 95% CI [− 2.80, − 2.01]. There was a significant effect of long-term relationship, *B* = − 0.62, *SE* = 0.25, *t* = − 2.49, *p* = .013, 95% CI [− 1.11, − 0.13]. Furthermore, short-term orientation had a significant effect, *B* = 0.78, *SE* = 0.21, *t* = 3.71, *p* < .001, 95% CI [0.37, 1.20]. No other significant effects occurred.

### Inter- and Intrasexual Differences in the Ten Marriage Criteria (E)

For the additional effects of long-term and short-term orientation and their interactions with sex and sexual orientation on the responses to the ten marriage criteria, see Table 5.

**Table 5:** Results of the binomial regression analyses to predict the preferences for the marriage criteria of lesbian women, gay men, and bisexual women and men

	… Who already was married	… Who has no employment	… Who has already children	…Who has another religion	…Who has another skin color	…Who earns much more	…Who earns much less	… Who does not look good	…Who has a higher education	…Who has a lower education
Sex	0.92 [0.67, 1.26]	**2.37** **[1.92, 2.94]**	**0.72** **[0.56, 0.93]**	**1.35** **[1.00, 1.81]**	**1.28** **[1.05, 1.55]**	0.50 [0.20, 1.26]	**3.63** **[2.82, 4.68]**	0.88 ]0.73, 1.06]	0.76 [0.51, 1.13]	**2.31** **[1.85, 2.90]**
Sexual Orientation (Sexor)	1.11 [0.81, 1.53]	**0.74** **[0.60, 0.92]**	1.11 [0.86, 1.44]	1.07 [0.80, 1.45]	0.83 [0.68, 1.01]	2.14 [0.85, 5.39]	**0.72** **[0.56, 0.92]**	0.99 [0.82, 1.19]	0.86 [0.58, 1.29]	1.06 [0.85, 1.32]
Short-term Orientation (STO)	1.19 [0.86, 1.65]	1.21 [0.96, 1.52]	1.09 [0.84, 1.40]	1.13 [0.85, 1.50]	**0.80** **[0.65, 0.99]**	0.57 [0.23, 1.40]	**1.38** **[1.09, 1.74]**	1.10 [0.90, 1.34]	1.05 [0.69, 1.58]	**1.25** **[1.00, 1.55]**
Long-term Orientation (LTO)	0.78 [0.53, 1.15]	1.20 [0.93, 1.55]	**0.61** **[0.44, 0.83]**	1.03 [0.71, 1.48]	1.29 [1.00, 1.66]	1.14 [0.67, 1.92]	0.94 [0.71, 1.25]	1.18 [0.93, 1.48]	0.74 [0.45, 1.20]	1.18 [0.90, 1.54]
Sex × LTO	1.28 [0.87, 1.86]	0.88 [0.69, 1.13]	1.24 [0.92, 1.66]	1.36 [0.95, 1.94]	1.18 [0.93, 1.50]	0.72 [0.43, 1.20]	0.93 [0.71, 1.22]	0.99 [0.79, 1.23]	1.28 [0.80, 2.05]	0.99 [0.77, 1.28]
Sex × STO	**1.38** **[1.00, 1.91]**	1.28 [1.02, 1.61]	0.84 [0.65, 1.09]	1.20 [0.90, 1.59]	1.02 [0.83, 1.25]	0.54 [0.22, 1.31]	1.09 [0.86, 1.37]	1.00 [0.83, 1.22]	1.32 [0.88, 1.97]	1.09 [0.88, 1.34]
Sex × Sexor	1.05 [0.77, 1.44]	1.00 [0.81, 1.23]	0.89 [0.69, 1.14]	1.03 [0.76, 1.38]	1.02 [0.84, 1.24]	1.53 [0.61, 3.83]	1.11 [0.87, 1.42]	0.93 [0.77, 1.11]	0.95 [0.64, 1.40]	0.94 [0.76, 1.17]
Sexor × LTO	0.69 [0.48, 1.01]	0.88 [0.69, 1.12]	0.85 [0.63, 1.15]	**0.69** **[0.49, 0.99]**	0.79 [0.62, 1.01]	0.84 [0.50, 1.40]	0.95 [0.72, 1.25]	0.94 [0.75, 1.17]	0.88 [0.55, 1.42]	1.19 [0.92, 1.53]
Sexor × STO	**0.65** **[0.47, 0.89]**	0.87 [0.70, 1.09]	0.92 [0.71, 1.17]	0.77 [0.58, 1.02]	0.93 [0.76, 1.14]	1.38 [0.57, 3.36]	0.97 [0.77, 1.21]	0.92 [0.76, 1.12]	0.94 [0.63 1.39]	0.90 [0.73, 1.10]
Sex × Sexor × STO	1.02 [0.74, 1.40]	0.80 [0.64, 1.00]	1.09 [0.85, 1.41]	0.95 [0.72, 1.26]	0.92 [0.75, 1.13]	**2.79** **[1.15, 6.79]**	0.82 [0.65, 1.02]	1.02 [0.84, 1.24]	0.78 [0.53, 1.17]	1.09 [0.88, 1.34]
Sex × Sexor × LTO	1.07 [0.73, 1.56]	1.14 [0.89, 1.45]	**0.71** **[0.53, 0.96]**	1.07 [0.75, 1.53]	0.98 [0.77, 1.25]	1.06 [0.64, 1.78]	1.18 [0.90, 1.56]	0.95 [0.76, 1.19]	1.07 [0.67, 1.71]	1.01 [0.78, 1.30]
Age	**0.93** **[0.90, 0.95]**	**1.02** **[1.00, 1.04]**	**0.92** **[0.90, 0.94]**	0.99 [0.97, 1.01]	**1.03** **[1.01, 1.04]**	1.02 [0.99, 1.06]	**1.06** **[1.04, 1.08]**	1.01 [0.99, 1.02]	1.02 [0.99, 1.06]	**1.04** **[1.03, 1.06]**
(Constant)	0.09	1.11	0.17	0.14	0.56	0.03	0.50	1.44	0.06	0.52

Sex (higher value for women) and sexual orientation (higher value for bisexual) are contrast-coded; LTO, STO, and age are mean-centered. Best predictors (largest predictive value and no CI overlap with other predictors) are printed bold

***p < .001, **p < .01, *p < .05

There were three significant two-way interactions. First, regarding the question if already being married is a deal-breaker, we found two significant interactions of short-term orientation with sex and sexual orientation. For men, there was no significant effect of short-term orientation for the willingness to marry that partner, *OR* = 0.86, 95% CI [0.55, 1.33], Wald *χ*^2^ = 0.46*, p* = .496, whereas for women, there was a significant effect, *OR* = 1.65, 95% CI [1.05, 2.60], Wald *χ*^2^ = 4.72*, p* = .030. For gay and lesbian people, there was a significant effect of short-term orientation*, OR* = 1.95, 95% CI [1.19, 3.18], Wald *χ*^2^ = 7.05*, p* = .008, but not for bisexual people, *OR* = 0.75, 95% CI [0.50, 1.13], Wald *χ*^2^ = 1.91*, p* = .167.

Second, regarding a partner with another religion, there was a significant sexual orientation × long-term relationship orientation. Analysis revealed that for gay/ lesbian people and bisexual people, the relationship of long-term relationship orientation was in the opposite direction, whereas also not significant. For gay and lesbian people, there was a (non-significant) positive relationship*, OR* = 1.62, 95% CI [0.87, 3.02], Wald *χ*^2^ = 2.28*, p* = .131, and a negative relationship for bisexual people, *OR* = 0.75, 95% CI [0.53, 1.08], Wald *χ*^2^ = 2.43*, p* = .119.

Additionally, we found two significant three-way interactions. First, for marrying someone who already has children, post-hoc analyses revealed no significant sex × long-term orientation interaction for bisexual people, *OR* = 0.92, 95% CI [0.67, 1.25], Wald *χ*^2^ = 0.30*, p* = .582, but for gay/lesbian people, *OR* = 1.69, 95% CI [1.03, 2.77], Wald *χ*^2^ = 4.34*, p* = .037. For gay men, there was a (non-significant) negative effect for long-term orientation, *OR* = 0.51, 95% CI [0.26, 1.01], Wald *χ*^2^ = 3.70*, p* = .054, and a positive (non-significant) effect for lesbian women, *OR* = 1.18, 95% CI [0.54, 2.58], Wald *χ*^2^ = 0.17*, p* = .684.

Second, for the partner who earns much more, there was a significant short-term orientation × sex × sexual orientation interaction. For bisexual people, there was no significant sex × short-term sexual orientation interaction, *OR* = 1.38, 95% CI [0.95, 2.01], Wald *χ*^2^ = 2.77*, p* = .096, whereas for gay/lesbian people, we found a significant interaction, *OR* = 0.41, 95% CI [0.20, 0.81], Wald *χ*^2^ = 6.42*, p* = .011. Short-term orientation had a significant positive effect for gay men, *OR* = 2.10, 95% CI [1.03, 4.30], Wald *χ*^2^ = 4.16*, p* = .041, but no significant effect for lesbian women, *OR* = 0.13, 95% CI [0.004, 4.01], Wald *χ*^2^ = 1.37*, p* = .241.

### Differences in the Domains of Mate Selection Preferences of Lesbian Women, Gay Men, Bisexual and Heterosexual Women and Men

Finally, we compared the mate selection preferences of the LGB sample with the heterosexual sample to answer H1. For direct comparison, we used the data set of self-identified heterosexual individuals reported in Schwarz and Hassebrauck (2012), and performed an Exploratory Factor Analysis with Promax rotation to extract domains of mate selection preferences in the heterosexual sample. The domains were comparable between the samples, but as there were slight differences between the domains of mate selection preferences of the LGB sample and the heterosexual sample, we took a closer look at the items which were identical in both the LGB and heterosexual samples (see the bold items in Table 1).

When comparing the factor loadings, we identified similar patterns in both the LGB and heterosexual samples to finally answer H1. The LGB sample's first factor, Factor I., the caring partner, was comparable to the care and understanding domain in the heterosexual sample. Factor II., adventurous partner, was similar to the preference for a dominant partner and social partner in the heterosexual sample. The third factor, Factor III., enlightened partner, was comparable to the preference for an intellectual partner in the heterosexual sample. Factor IV., cultivated partner, in the LGB sample was comparable to the domain of cultivated partner in the heterosexual sample. Factor V., preference for a physically attractive partner, and Factor VI., preference for a wealthy and generous partner, fitted the domains in the heterosexual sample. Factor VII., an approachable partner, was also represented in the heterosexual sample. Factor VIII., a comedic partner, matched the preference for a humorous partner in the heterosexual sample, while Factor IX., a domestic partner, matched the creative and domestic domain in the heterosexual sample. The preference for a like-minded partner (Factor X.) matched the preferences of the heterosexual sample perfectly. Factor XI., preference for a child-friendly partner, included two items represented in the heterosexual sample.

We computed new unit-weighted scales using the items which were represented in both the LGB and heterosexual samples (see the bold items in Table 1), and conducted several robust regression analyses with the scale's mean as the dependent variable, and used sexual orientation (reference category heterosexual vs. lesbian/gay, and reference category heterosexual vs. bisexual) and sex (0 = men, 1 = women) as predictors (see Table 6 for descriptive statistics). Age was a covariate in all analyses.

**Table 6:** Descriptive statistics for differences in the domains of mate selection preferences of lesbian women, gay men, bisexual and heterosexual women and men

Domains of mate selection preference	Heterosexual men		Heterosexual women		Gay men		Lesbian women		Bisexual men		Bisexual women	
	*M*	*SD*	*M*	*SD*	*M*	*SD*	*M*	*SD*	*M*	*SD*	*M*	*SD*
I. Caring	4.32	0.47	4.50	0.43	4.27	0.62	4.53	0.45	4.27	0.58	4.43	0.51
II. Adventurous	3.54	0.53	3.78	0.49	3.71	0.62	3.87	0.53	3.62	0.59	3.82	0.61
III. Enlightened	3.44	0.61	3.74	0.61	3.38	0.77	3.66	0.62	3.48	0.74	3.69	0.69
IV. Cultivated	3.76	0.62	4.00	0.57	3.83	0.72	4.05	0.61	3.88	0.80	3.92	0.70
V. Physically attractive	3.87	0.70	3.61	0.72	3.98	0.76	3.67	0.73	3.98	0.71	3.70	0.79
VI. Wealthy and generous	2.42	0.70	3.00	0.77	2.74	0.83	3.05	0.83	2.60	0.83	2.97	0.85
VII. Approachable	4.02	0.60	4.07	0.63	4.14	0.65	4.17	0.64	4.10	0.64	4.02	0.72
VIII. Comedic	3.91	0.68	4.09	0.68	4.01	0.72	4.16	0.62	3.95	0.75	4.12	0.75
IX. Domestic	2.86	0.72	2.75	0.73	3.07	0.88	2.91	0.82	3.04	0.87	2.86	0.86
X. Like-minded	3.77	0.67	3.99	0.61	3.73	0.86	4.11	0.61	3.90	0.78	3.91	0.75
XI. Child-friendly	3.22	1.08	3.13	1.16	3.12	1.35	3.38	1.17	3.15	1.14	3.21	1.25

We used items which were represented in both the LGB (*n* = 710) and heterosexual (*n* = 21,245) sample (after conducting an EFA with Promax rotation in both LGB and heterosexual sample) to compute the new unit-weighted scales

In all eleven robust regression analyses (see Table 7), the sex of the participants was a significant predictor. There were significant effects of the lesbian/ gay sexual orientation in four domains (II. adventurous partner, VI. wealthy and generous partner, VII. approachable partner, XI. child-friendly partner), and the bisexual orientation was a significant predictor for six domains (II. adventurous partner, IV. cultivated partner, V. physical attractive partner, VIII. comedic partner, IX. domestic partner, X. like-minded partner).

**Table 7:** Results of the regression analyses to predict differences in the common domains of mate selection preferences of lesbian women, gay men, bisexual and heterosexual women and men

Domains of mate selection preference	Sex of participants	Sexual Orientation: Heterosexual versus lesbian/gay	Sexual Orientation: Heterosexual versus bisexual	Sex × Sexual Orientation: Heterosexual versus lesbian/gay	Sex × Sexual Orientation: Heterosexual versus bisexual	Age
I. Caring	0.17*** **[0.16, 0.18]**	0.22 [− 0.06, 0.11]	0.01 [− 0.06, 0.08]	0.05 [− 0.06, 0.17]	− 0.04 [− 0.12, 0.04]	0.003*** [0.002, 0.003]
II. Adventurous	0.23*** **[0.22, 0.25]**	0.15** **[0.04, 0.26]**	0.11* [0.02, 0.19]	− 0.02 [− 0.16, 0.12]	− 0.03 [− 0.14, 0.07]	− 0.001*** [− 0.002, − 0.001]
III. Enlightened	0.30*** **[0.28, 0.32]**	− 0.05 [− 0.19, 0.08]	0.08 [− 0.03, 0.18]	0.01 [− 0.16, 0.18]	− 0.08 [− 0.20, 0.05]	− 0.01*** [0.01, 0.01]
IV. Cultivated	0.23*** **[0.22, 0.25]**	0.12 [− 0.001, 0.24]	0.20*** **[0.11, 0.30]**	− 0.03 [− 0.19, 0.13]	− 0.22*** **[**− **0.34, − 0.11]**	0.01*** [0.01, 0.01]
V. Physically attractive	− 0.27*** **[**− **0.29,** − **0.25]**	0.11 [− 0.04, 0.25]	0.14* [0.02, 0.25]	− 0.06 [− 0.25, 0.12]	− 0.04 [− 0.17, 0.10]	− 0.01*** [− 0.01, − 0.01]
VI. Wealthy and generous	0.59*** **[0.57, 0.61]**	0.32*** [0.16, 0.48]	0.12 [− 0.01, 0.24]	− 0.21 [− 0.42, 0.002]	− 0.15 [− 0.30, 0.004]	0.01*** [0.01, 0.01]
VII. Approachable	0.05*** **[0.03, 0.06]**	0.13* **[0.01, 0.26]**	0.08 [− 0.01, 0.18]	− 0.04 [− 0.20, 0.12]	− 0.11 [− 0.22, 0.01]	0.001 [− 0.0001, 0.001]
VIII. Comedic	0.18*** **[0.16, 0.19]**	0.07 [− 0.07, 0.20]	0.12* **[0.02, 0.23]**	− 0.04 [− 0.21, 0.14]	− 0.08 [− 0.21, 0.05]	− 0.01*** [− 0.01, − 0.01]
IX. Domestic	− 0.13*** **[**− **0.15, − 0.11]**	0.16 [− 0.004, 0.32]	0.17** **[0.04, 0.29]**	0.05 [− 0.16, 0.26]	− 0.05 [− 0.20, 0.10]	0.01*** [0.01, 0.01]
X. Like-minded	0.20*** **[0.18, 0.21]**	0.05 [− 0.08, 0.18]	0.17** **[0.07, 0.27]**	0.08 [− 0.09, 0.25]	− 0.19** **[− 0.31,** − **0.07]**	0.003*** [0.003, 0.004]
XI. Child-friendly	− 0.08*** [− 0.11, − 0.05]	− 0.32** **[− 0.54, − 0.11]**	0.13 [− 0.04, 0.30]	0.34* **[0.05, 0.62]**	− 0.19 [− 0.40, 0.02]	− 0.06*** [− 0.06, − 0.05]

LGB: *n*_*1*_ = 710, Heterosexual: *n*_*2*_ = 21,254. Age served as a covariate in the analyses. Sex is dummy-coded. Best predictors (largest predictive value and no CI overlap with other predictors) are printed bold

***p < .001, **p < .01, *p < .05

The sex × bisexual orientation interaction was a significant predictor for two domains (IV. cultivated partner and X. like-minded partner). For the Factor IV. (cultivated), post-hoc analysis revealed a significant sex difference for heterosexual people, *B* = 0.23, *SE* = 0.01, *t* = 28.53, *p* < .001, 95% CI [0.22, 0.25], but no sex differences were shown for bisexual people, *B* = 0.05, *SE* = 0.07, *t* = 0.66, *p* = .507, 95% CI [− 0.10, 0.19]. For Factor X., a like-minded partner, the analysis revealed a significant sex difference for heterosexual people, *B* = 0.20, *SE* = 0.01, *t* = 22.71, *p* < .001, 95% CI [0.18, 0.21], but no sex differences were found for bisexual people, *B* = 0.03, *SE* = 0.45, *p* = .653, 95% CI [− 0.11, 0.18].

There was one significant sex × gay/lesbian interaction for Factor XI., a child-friendly partner. Further analyses revealed a significant sex difference for heterosexual people, *B* = − 0.08, *SE* = 0.02, *t* = − 5.22, *p* < .001, 95% CI [− 0.11, − 0.05], but no sex differences were found for gay/lesbian people, *B* = 0.26, *SE* = 0.18, *t* = 1.44, *p* = .153, 95% CI [− 0.09, 0.61].

Again, we considered the 95% confidence interval to identify the strongest predictor (printed in bold in Table 7). If two predictors overlap between the CI, then both are seen as equally strong. In ten of eleven analyses, the sex of participants was a strong predictor, and in four models, the sex of participants alone was the best predictor of mate preferences.

### Differences in Age Acceptance of Lesbian Women, Gay Men, Bisexual and Heterosexual Women and Men (H5)

We analyzed differences in the accepted maximum and minimum age of a potential partner (H5) by calculating two robust regressions using the “robust” package, with sex (0 = men, 1 = women) and sexual orientation (reference category heterosexual vs. lesbian/gay, and reference category heterosexual vs. bisexual) and participants' age (as a covariate), on the maximum and the minimum partner’s age. There were 54 missing responses for the minimum age, so the analysis was conducted with *n* = 21,901, and as there were 39 missing responses for the maximum age, the analysis was conducted with *n* = 21,916.

For the maximum age (*R*^2^ = .288), sex has a significant effect, *B* = 4.12, *SE* = 0.04, *t* = 92.10, *p* < .001, 95% CI [4.03, 4.21]. Women (*M* = 8.30, *SD* = 6.07) accepted much older partners than men (*M* = 4.53, *SD* = 6.89). Lesbian/gay orientation did not differ from heterosexual orientation, *B* = − 0.33, *SE* = 0.35, *t*(21,909) = − 0.92, *p* = .36, 95% CI [− 1.02, 0.37], but bisexuals were less restrictive in their age acceptance than heterosexuals, *B* = 0.91, *SE* = 0.28, *t* = 3.29, *p* = .001, 95% CI [0.37, 1.45]. The participants' age had a significant effect, *B* = − 0.09, *SE* = 0.002, *t* = − 45.44, *p* < .001, 95% CI [− 0.10, − 0.09]. Sex and sexual orientation did not interact significantly.

For the minimum age (*R*^2^ = .332), men (*M* = 10.02, *SD* = 4.79) accepted a much younger partner than women (*M* = 4.93, *SD* = 3.43), *B* =  − 4.64, *SE* = 0.05, *t* = − 101.26, *p* < .001, 95% CI [− 4.73, − 4.55]. Participants' age also had a significant effect here, *B* = 0.14, *SE* = 0.002, *t* = 68.15, *p* < .001, 95% CI [0.14, 0.15], but not the lesbian/gay orientation, *B* = − 0.65, *SE* = 0.36,* t* = − 1.83, *p* = .067, 95% CI [− 1.34, 0.05], or the bisexual orientation, *B* = 0.10, *SE* = 0.29,* t* = 0.34, *p* = .732, 95% CI [− 0.47, 0.67]. There was a significant interaction of sex and lesbian/ gay orientation, *B* = 1.04, *SE* = 0.46, *t* = 2.28, *p* = .023, 95% CI [0.15, 1.93].

Simple slope analyses revealed that bisexual women would accept an even younger partner than heterosexual women (*M* = 4.92, *SD* = 3.41), *B* = 0.46, *SE* = 0.18, *t* = 2.61, *p* = .009, 95% CI [0.12, 0.80], whereas for men there was no difference in the minimum age acceptance, *B* = 0.32, *SE* = 0.35, *t* = 0.03, *p* = .352, 95% CI [− 0.361, 1.10].

### Differences in the Ten Marriage Criteria of Lesbian Women, Gay Men, Bisexual and Heterosexual Women and Men

Differences in the responses to the ten marriage criteria (adopted from Sprecher et al., 1994) depending on sexual orientation were analyzed with several robust binomial regression analyses (using the “robustbase” package for R (Maechler et al., 2021; Todorov & Filzmoser, 2009) with sex (0 = men, 1 = women) and sexual orientation (reference category heterosexual vs lesbian/gay, and reference category heterosexual vs bisexual), their interaction as predictors and age of the participants as a covariate. We used odds ratios (OR) for the interpretation of the results. For the sex differences, an OR greater than 1 indicates a higher probability that women will respond to the question with “no”, whereas an OR lower than 1 indicates that men are more likely to respond to the question with “no”. Additionally, for sexual orientation, an OR greater than 1 indicates a higher probability that lesbian women, gay men, or in the other case, bisexuals responded to the question with “no”. The number of “Yes” answers to these questions for each group can be seen in Table 8. The robust regression models are summarized in Table 9.

**Table 8:** Descriptive statistics of preferences for marriage criteria of lesbian women, gay men, bisexual and heterosexual women and men

“Could you imagine marrying someone …”	%—Yes response					
	Heterosexual men	Heterosexual women	Gay men	Lesbian women	Bisexual men	Bisexual women
… Who has no employment	75.3	28.0	64.2	30.8	72.0	34.8
… Who has already had children	81.3	86.4	73.7	82.3	79.6	85.1
… Who has another religion	91.0	84.5	90.5	86.2	89.8	82.9
… Who already was married	91.9	91.8	86.3	90.8	91.7	88.1
… Who has another skin color	74.1	51.8	68.4	52.3	72.0	61.3
… Who earns much more	92.8	95.8	92.6	96.2	91.1	94.5
… Who earns much less	91.0	37.9	83.2	42.3	84.7	43.3
… Who does not look good	35.2	47.7	38.9	43.1	36.3	46.6
… Who has a higher education	94.9	96.3	92.6	96.2	91.1	96.6
… Who has a lower education	87.4	43.0	82.1	51.5	76.4	52.7

Answers given on a dichotomous "yes" or "no" scale

**Table 9:** Results of the binomial regression analyses to predict preferences for marriage criteria of lesbian women, gay men, bisexual and heterosexual women and men

“Could you imagine marrying someone …”	Sex of participants	Sexual Orientation: Heterosexual versus lesbian/gay	Sexual Orientation: Heterosexual versus bisexual	Sex × Sexual Orientation: Heterosexual versus lesbian/gay	Sex × Sexual Orientation: Heterosexual versus bisexual	Age
… Who has no employment	7.88*** **[7.40, 8.39]**	1.79** **[1.17, 2.75]**	1.14 [0.80, 1.62]	0.51* **[0.29, 0.90]**	0.66 [0.44, 1.02]	1.01*** **[1.01, 1.02]**
… Who has already had children	0.70*** **[0.64, 0.76]**	0.82 [0.47, 1.43]	1.50 [0.95, 2.38]	1.02 [0.48, 2.19]	0.43** **[0.24, 0.78]**	0.90*** **[0.90, 0.90]**
… Who has another religion	1.86*** **[1.70, 2.04]**	1.02 [0.39, 2.11]	1.19 [0.69, 2.06]	0.83 [0.34, 2.03]	0.92 [0.49, 1.71]	**0.99** **[0.99, 0.99]**
… Who already was married	1.08 [0.96, 1.21]	0.77 [0.35, 1.69]	1.32 [0.65, 2.71]	0.77 [0.27, 2.22]	0.64 [0.28, 1.46]	**0.88** **[0.88, 0.89]**
… Who has another skin color	2.70*** **[2.55, 2.87]**	1.44 [0.93, 2.23]	1.04 [0.72, 1.48]	0.74 [0.42, 1.29]	0.70 [0.46, 1.06]	1.02*** **[1.02, 1.02]**
… Who earns much more	0.55*** **[0.48, 0.63]**	1.17 [0.51, 2.68]	1.16 [0.65, 2.09]	1.00 [0.28, 3.50]	1.16 [0.52, 2.60]	1.03*** **[1.02, 1.04]**
… Who earns much less	17.53*** **[16.09, 19.09]**	2.47** **[1.42, 4.30]**	1.70* **[1.08, 2.68]**	0.38** **[0.20, 0.74]**	0.51** **[0.31, 0.85]**	1.03*** **[1.03, 1.03]**
… Who does not look good	0.60*** **[0.56, 0.63]**	0.86 [0.57, 1.30]	0.95 [0.68, 1.32]	1.41 [0.82, 2.43]	1.19 [0.87, 1.64]	1.00 [0.99, 1.00]
… Who has a higher education	0.68*** **[0.59, 0.79]**	1.86 [0.83, 4.20]	1.64 [0.91, 2.96]	0.58 [0.15, 2.22]	0.54 [0.21, 1.39]	1.04*** **[1.03, 1.05]**
… Who has a lower education	10.12*** **[9.37, 10.92]**	1.95* **[1.13, 3.36]**	1.98*** **[1.34; 2.91]**	0.42** **[0.22, 0.81]**	0.48** **[0.31, 0.75]**	1.04*** **[1.04, 1.04]**

Answers given on a dichotomous yes or no scale. Odds ratio and 95% confidence interval for Odds ratios are presented. Sex is dummy-coded (higher values for women), age is mean-centered

Significant results are printed bold

***p < .001, **p < .01, *p < .05

Similarly to the previous analyses, the sex of participants was a strong predictor in nine out of ten models. Significant differences between heterosexuals and gay men/lesbian women were identified in three models, and in two models, significant differences between heterosexuals and bisexuals emerged. Moreover, six significant interactions between sex and sexual orientation were found in four domains.

When taking a closer look at financial aspects, we found significant differences. More men than women and more heterosexual people than lesbian or gay people could imagine marrying someone unemployed, whereas there was no difference for bisexual people. Additionally, there was a significant interaction of sex and lesbian/gay sexual orientation. Simple slope analyses revealed that for women, there was no significant difference between heterosexual and lesbian women, *OR* = 0.93, 95% CI [0.64, 1.36], z = − 0.36, *p* = .718, whereas more heterosexual men than gay men could imagine marrying an unemployed partner, *OR* = 1.75, 95% CI [1.15, 2.67], *z* = 2.59, *p* = .010. More women than men would marry a partner who earns much more than they do. However, more men than women could imagine marrying someone who earns much less than they do. Additionally, there was a significant effect for bisexual and lesbian/ gay people. A significant interaction of sex and lesbian/ gay and bisexual orientation qualified these effects. Heterosexual and lesbian women, *OR* = 0.94, 95% CI [0.66, 1.34], z = − 0.34, *p* = .737, and bisexual women, *OR* = 0.88, 95% CI [0.70, 1.09], *z* = − 1.14, *p* = .254, did not differ in their responses. There was a significant difference between heterosexual men and gay men, *OR* = 2.21, 95% CI [1.28, 3.82], *z* = 2.86, *p* = .004, as well as bisexual men, *OR* = 1.71, 95% CI [1.10, 2.65], *z* = 2.37, *p* = .018.

When considering a potential partner's education level, more women than men could imagine marrying a partner with a higher level of education than themselves. For the lower education, there was a significant difference between men and women, and heterosexual and lesbian/gay people as well as heterosexual and bisexual people. Additionally, there was a significant interaction for sex and both sexual orientations. For women, there was no significant difference between heterosexual and lesbian women, *OR* = 0.83, 95% CI [0.58, 1.19], *z* = − 1.02, *p* = .308, and bisexual women, *OR* = 0.96, 95% CI [0.76, 1.20], *z* = − 0.37, *p* = .713. Also, there was no significant difference for heterosexual men and gay men, *OR* = 1.64, 95% CI [0.97, 2.79], *z* = 1.83, *p* = .068, but significant for bisexual men, *OR* = 2.02, 95% CI [1.39, 2.94], *z* = 3.67, *p* < .001.

More women than men could imagine marrying a partner who already has children. Additionally, there was a significant difference for heterosexual and bisexual people, which was qualified by a sex × bisexual orientation interaction, *OR* = 0.47, 95% CI [0.27, 0.80], *z* = − 2.76, *p* = .006. For women, there was no significant difference between heterosexual and lesbian women, *OR* = 0.86, 95% CI [0.52, 1.42], *z* = − 0.59, *p* = .555, nor bisexual women, *OR* = 0.74, 95% CI [0.53, 1.03], *z* = − 1.77, *p* = .077. For men, there was no difference between heterosexual and gay men, *OR* = 1.11, 95% CI [0.67, 1.82], *z* = 0.40, *p* = .692, but there was a significant difference for bisexual men and heterosexual men, *OR* = 1.58, 95% CI [1.03, 2.40], *z* = 2.11, *p* = .035.

Considering the potential partner's physical attributes, more men than women could imagine marrying a partner with a different skin color. Also, more women than men could imagine marrying someone who is not good-looking, and for this criterion, there were no more differences according to sexual orientation.

In nine out of ten models, the age of participants had, albeit weak, effects. The older the participants, the more they could imagine marrying a partner who was already married before. The older the participants, the less they could imagine marrying someone unemployed. However, with increasing age, more participants could imagine marrying someone who already has children. With increasing age, more participants could also imagine marrying a partner with different religious beliefs but could not imagine marrying someone with different skin color. They wanted to marry someone who earns the same as they do. Older participants could not imagine marrying a partner with a higher level of education than themselves, and they could also not imagine marrying someone with lower level of education than themselves. Interestingly, marrying a partner who does not look good did not covary with age. Marrying someone who does not look good seems to be a deal-breaker (especially for men) across the lifespan.

## Discussion

We examined the domains of mate selection preferences for a potential long-term partner in a sample of lesbian women, gay men, bisexual women and men (LGB) with a focus on intersexual (between-sex) and intrasexual (within-sex) mating strategies differences. Moreover, we compared the importance of these domains when selecting potential long-term partners between lesbian women, gay men, bisexual and heterosexual women and men. Given the number of participants (710 LBG and 21,245 heterosexual), the number of variables (82 mate selection criteria for a long-term partner, two questions regarding minimum and maximum acceptable age, ten marriage criteria of a potential partner), the number of sexual orientations (lesbian women, gay men, bisexual women and men in comparison to heterosexual men and women), as well our focus on intersexual and intrasexual variations, this study is a comprehensive study on partner preferences in LGB people compared to the already well-known partner preferences of heterosexual people.

Eleven domains of mate selection preferences were extracted based on this large sample of lesbian, gay, and bisexual single participants searching online for a potential long-term partner. Some of the domains extracted from our data are comparable to those from Regan and colleagues (2001); notably, our domain of preferences for a wealthy and generous, physically attractive, enlightened, and caring partner are comparable to the social status, physically appealing, intellect and interpersonal sensitivity domains. Also, some common domains were seen in both heterosexual and LGB participants (e.g., wealthy and generous, physically attractive partner), and a similar pattern of mate selection preferences was reflected across sexual orientations. The items included in the domains of a caring partner are somewhat comparable to the sincerity domain, which prior research examined in partner preferences of lesbian women and gay men with mixed results. Some personal ad studies found that women (Deaux & Hanna, 1984; Gonzales & Meyers, 1993) and especially heterosexual women (Deaux & Hanna, 1984), sought a sincere partner, while other studies found that especially lesbian women sought a sincere partner more than heterosexual women (Smith et al., 2011). Other studies did not find the effects of sexual orientation (Gonzales & Meyers, 1993). Our results seem to favor a main effect of sex, i.e., women value a sincere partner more than men.

Our results revealed more sex differences between lesbian women, gay men, and bisexual women and men. In the LGB sample, men compared to women showed a stronger preference for a physically attractive partner and a domestic partner, replicating prior findings in the literature on lesbian women, gay men, and heterosexual women and men (Ha et al., 2012; Lippa, 2007). Women showed a stronger preference for six characteristics, and the largest effect size for sex differences could be identified for preferences for a wealthy and generous partner. These findings are robust in most of the mate selection preference literature, although almost all studies focused on heterosexual participants.

An interesting finding is the importance of intrasexual differences in our sample of lesbian women, gay men, and bisexual women and men. As mentioned, long-term orientation is an important variable in all eleven domains of mate selection preferences. The role of differences in long-term orientation is especially highlighted for a caring, domestic, child-friendly partner and a cultivated partner. This result is comparable to results in heterosexual samples, in which long-term orientation is strongly associated with related domains (see Study 1, Schwarz et al., 2020). Differences in the long-term and short-term relationship orientation both predicted the preference for a physically attractive partner. This result is in line with prior research, which shows that for short (mostly sexual) encounters, the physical attractiveness of a potential partner is important, and, although it is more pronounced for men, women show a higher preference for physical attributes in short-term contacts. This trade-off is a result that can be identified in both women and men with different sexual orientations (Li et al., 2002; Regan et al., 2001; Schwarz et al., 2020). Short-term orientation was a significant predictor in preference for a wealthy and generous partner, although not the strongest. This result fits the idea that short-term mating allows people to extract immediate financial resources from a potential partner (e.g., resource accrual hypothesis; Symons, 1979).

For the ten marriage criteria, our results are also in line with prior research. For example, heterosexual men indicated that they would be more likely to marry a partner who earns much less than they do more than bisexual men, and more than gay men. However, only bisexual and heterosexual men differed in their preferences for a partner who had a lower education, but there was no difference between gay and heterosexual men. This is not in line with prior research which indicates that gay men showed a higher preference for partners that have finished their education in comparison to heterosexual men (Ha et al., 2012).

Our results regarding the accepted minimum and maximum age of lesbian women, gay men, and bisexual women and men are almost comparable to results from heterosexual samples (Schwarz & Hassebrauck, 2012) and results from prior research that looked at age preferences across different sexual orientations. For the minimum age, there was a sex difference in that women accepted much older partners than men. Gay/ lesbian and heterosexual people showed no significant differences in their age acceptance, but bisexual people showed a slightly greater difference between their age and their partner's age. We found no interaction effects of sex and sexual orientations, indicating no further differences between lesbian, bisexual and heterosexual women and men. This is somewhat controversial to prior literature, in which it was, for example, found that gay men and lesbian women showed higher maximum age and acceptable age range tolerance than heterosexual men and women (Conway et al., 2015; Kenrick et al., 1995). However, these studies only considered lesbian women (for recent data on the youngest and oldest considered sex partner and sexual orientation cf. Antfolk, 2017). Moreover, it should be kept in mind that the mating market in the LGB community is relatively smaller than for heterosexuals. Thus, greater flexibility in age preferences might be rather a psychological response to accommodate the constraints of limited available potential partners. Further studies could test this idea directly when comparing the age range of participants from larger vs. smaller LGB communities. We would predict that living in smaller LGB communities could contribute to accepting an even wider age range compared to living in larger LGB communities. Unfortunately, our data do not allow us to test such contextual effects on age preferences.

From an evolutionary perspective, the potential partner's age signals its mate value and gives important cues about its reproductive success. As such, men mostly prefer younger but already fertile women because females' reproductive value decreases with age. For women, resource productivity increases with males' age. This means that they usually prefer older to younger men. Additionally, a men's reproductive value is less closely linked to age than it is for women (Conroy-Beam & Buss, 2019).

Our results support the notion in prior literature that the sex of participants is an important factor when considering partner preferences and mating strategies. As prior research claims, the effects of sex differences in the pattern of mate preferences are eight times larger than in other psychological studies, and mate preferences are “dramatically different for the sexes” (Conroy-Beam et al., 2015, p. 1090). Although there are some differences in the preferences of LGB and heterosexual men and women, the differences according to sexual orientation are relatively small. The comparable pattern suggests that the processes underlying the adaptions of our evolutionary history were similar across humans (Howard et al., 1987), and that some of the (cognitive) processes in mating depend on the biological sex (Howard & Perilloux, 2017; Rinn et al., 2020). The modularity hypothesis (Cosmides & Tooby, 1987; Fodor, 1983) states that modules are a set of psychological mechanisms and can operate independently, which aligns with these findings. According to this theory, differences in one module, like sexual orientation, do not directly lead to differences in another module, like partner preferences (Ha et al., 2012).

### Limitations and Future Research

One critical aspect of our research was the focus on long-term relationships, and we did not differentiate between preferences for a short-term or a long-term partner. As there are somewhat different patterns in mating preferences depending on the time frame of the potential relationship (Gobrogge et al., 2007; Schwarz et al., 2020), it would also be important to analyze preferences for short-term partners in LGB mate selection preferences.

A second important aspect focused on the measurement of sexual orientation. We used only a categorical way of measuring sexual orientation, asking participants to choose from limited options (Sell, 1997). This categorical assessment has been criticized for several issues (for an overview cf. Salomaa & Matsick, 2019). In this study, the measurement was advantageous when comparing our results with prior research into mate selection preferences, but future research should include a different way of measuring sexual orientation. A measurement of sexual orientation should not only include categorical labeling but a continuum scale and assessments of attitudes, desire, and current, past, and future sexual experiences with same-sex or opposite-sex partners (e.g., via the Klein Sexual Orientation Grid; Klein et al., 1985; for a recent review see Salomaa & Matsick, 2019). It is crucial to distinguish between sex and gender, as well as sexual identities, preferences, and behaviors, as some individuals display consistent sexualities while others have varying experiences throughout their life course (Frederick et al., 2023). In line with a different perspective on sexual orientation, important subtypes of sexual orientation should also be included (e.g., gender roles, femme, and butch in lesbian female participants, Hiestand & Levitt, 2005).

Third, we did not assess the ethnicity in our sample. Compared to common US classifications of ethnicity, we can only speculate, based on our experience, that almost all participants were “White”. However, especially the finding that men in this sample seem to imagine having a partner with a different skin color than women should not be overinterpreted, as we do not know for sure the ethnicity of our sample.

Fourth, one should keep in mind that our sample is, at least compared to many other studies in the literature which rely on student age samples (cf. Schwarz & Hassebrauck, 2012) older (mean age around 39 years). On the one hand, this allowed us even better to compare our data with the heterosexual data from Schwarz and Hassebrauck (2012), with a mean age of around 41 years. However, on the other hand, our sample size was too restricted to analyze potential age effects in partner preferences directly. From our perspective, especially bisexual women should deal with, similar to heterosexual women or even stronger, trade-offs in their reproductive decisions in their life-span (especially pre- vs. postmenopausal), which could impact partner preferences. Further research, preferably longitudinal studies regarding the effects of age on partner preferences, especially in bisexual women, are needed to address this open question directly.

Finally, there are important trade-off effects when it comes to partner preferences. In real-life settings, people cannot always get what they want, and when participants can only make limited choices (e.g., have limited mating dollars), interesting results were found (Li et al., 2002). Future research should incorporate different methods to examine mate selection preferences, not only when analyzing sexual minorities.

### Conclusion

Our study has the advantage of considering more than a handful of mate selection criteria (84 characteristics) in a considerable sample of LGB men and women (*n* = 710). We compared these preferences with heterosexual participants (*n* = 21,245) by looking at the common preference characteristics. Lesbian women, gay men, bisexual and heterosexual women and men answered the same questions about their preferences for several characteristics of a potential long-term partner. As such, we could directly compare the similarities and differences without relying solely on qualitative reports from prior research. In addition, we considered interindividual differences in short-term and long-term relationship orientation. Although much is known about differences in preferences for short-term and long-term mating, almost all results are taken from heterosexual samples and consider female-male mating contexts.

Although we have found comparable patterns of both mate selection preferences and effects of short-term and long-term relationship orientation in lesbian women, gay men, bisexuals, and heterosexuals, this is not a reason to only include one sexual orientation in research. These results should prevent participants’ exclusion in samples and facilitate an inclusive research practice for examining partner preferences and mating strategies across sexual majorities and minorities.

## Data Availability

All data and material are available from the corresponding author upon request.
